# pH-responsive Zn^2+^-coordinated gellan gum/γ-polyglutamic acid hydrogel microspheres as functional biomaterials for intra-articular delivery: physicochemical design, smart release, and chondroprotective performance in osteoarthritis

**DOI:** 10.3389/fbioe.2026.1924086

**Published:** 2026-09-29

**Authors:** Kaiming Zhang, Haonan Xue, Xiangwei Li, Fuzheng Zou, Keyu Wei, Mao Nie, Xianding Sun

**Affiliations:** 1 Department of Orthopedic Surgery, The Second Affiliated Hospital of Chongqing Medical University, Chongqing, China; 2 Chongqing Municipal Clinical Research Center for Geriatrics, The Second Affiliated Hospital of Chongqing Medical University, Chongqing, China; 3 Department of Orthopedics, The Third Affiliated Hospital of Chongqing Medical University, Chongqing, China; 4 Tissue Repairing and Biotechnology Research Center, The Third Affiliated Hospital of Chongqing Medical University, Chongqing, China

**Keywords:** cartilage, hydrogel microspheres, osteoarthritis, PI3K-AKT, Zn^2+ ^coordination

## Abstract

**Introduction:**

Osteoarthritis (OA) is a degenerative joint disease characterized by progressive degradation of the articular cartilage matrix, pain-related functional impairment, and a paucity of effective disease-modifying therapies. Curculigoside (Cur), a bioactive compound isolated from Curculigo orchioides, possesses anti-inflammatory and tissue-protective properties; however, its direct intra-articular application is hampered by insufficient local retention and limited sustained action.

**Methods:**

We developed a Zn^2+^-coordinated pH-responsive hydrogel microsphere delivery system (PGG-Zn@Cur) based on gellan gum (GG) and γ-polyglutamic acid (γ-PGA) to enable localized intra-articular delivery of Cur. Its cytocompatibility and chondroprotective effects were evaluated in IL-1β-stimulated chondrocytes. Transcriptomic analysis was further performed to investigate the potential molecular mechanisms underlying its therapeutic effects.

**Results:**

PGG-Zn@Cur exhibited excellent cytocompatibility and restored cartilage matrix metabolic homeostasis in IL-1β-stimulated chondrocytes, as evidenced by increased COL2A1 and ACAN expression and reduced MMP13, ADAMTS5, and iNOS levels. Transcriptomic analysis implicated PI3K-AKT signaling and apoptosis-related pathways in the chondroprotective effects mediated by PGG-Zn@Cur. Mechanistically, PGG-Zn@Cur suppressed PI3K-AKT pathway hyperactivation, downregulated Cleaved Caspase-3 and Bax expression, attenuated chondrocyte apoptosis, and alleviated cartilage degeneration.

**Discussion:**

These findings establish PGG-Zn@Cur as a microenvironment-responsive intra-articular platform that maintains cartilage matrix homeostasis and alleviates PI3K-AKT-associated apoptosis-related responses, offering a promising strategy for disease-modifying OA therapy.

## 1 Introduction

As a widespread degenerative joint disorder, osteoarthritis (OA) imposes a considerable global health burden by causing persistent pain, limitations in mobility, ear-and-tear disorder, it is now recognized as a whole-joint disease involving articular cartilage, synovium, subchondral bone, meniscus, infrapatellar fat pad, and pain-related neuroimmune responses (Wang M. et al., 2022; Feng et al., 2026). During OA progression, inflammatory mediators and mechanical stress disrupt chondrocyte homeostasis, resulting in extracellular matrix degradation, loss of type II collagen and aggrecan, upregulation of catabolic enzymes such as MMP13 and ADAMTS5, and progressive cartilage structural deterioration (Kumavat et al., 2021; Yao et al., 2023). Current therapeutic options, such as non-steroidal anti-inflammatory drugs, intra-articular corticosteroid injections, hyaluronic acid supplementation, and joint replacement for advanced disease, are primarily aimed at symptom control rather than preventing cartilage deterioration or re-establishing extracellular matrix homeostasis (Jin et al., 2024; Bharadwaj et al., 2025; Wu et al., 2026; Zhang X. et al., 2026). Therefore, developing local therapeutic strategies that can maintain chondrocyte function, reduce inflammatory-catabolic injury, and achieve sustained action within the joint cavity remains an important challenge for OA intervention.

Curculigoside (Cur) is a naturally occurring phenolic glycoside isolated from Curculigo orchioides and has been increasingly investigated for its multiple biological activities, including anti-inflammatory, antioxidant, anti-apoptotic, and tissue-protective effects (Lei et al., 2025). Accumulating evidence indicates that Cur modulates NF-κB, JAK/STAT, and Nrf2 signaling pathways, alongside apoptosis-related cascades, implicating its therapeutic potential in inflammatory and tissue-degenerative conditions (Xu et al., 2023). These findings collectively position Cur as a promising candidate for OA therapy. However, like many natural small-molecule compounds, its direct administration is hampered by unfavorable drug-like properties, including poor water solubility, rapid *in vivo* clearance, low bioavailability, restricted local retention, and insufficient tissue-targeted accumulation (Park et al., 2024; Öztürk et al., 2024). Such shortcomings are especially detrimental in the context of intra-articular delivery, where premature clearance from the joint cavity and uncontrolled drug exposure markedly compromise the effective therapeutic duration (Gambaro et al., 2021). Consequently, the rational design of a localized delivery platform is imperative to prolong intra-articular residence of Cur and enable sustained, microenvironment-adaptive release.

Hydrogel provide a promising platform for intra-articular drug delivery because of their high water content, tissue-like physicochemical properties, injectability, and capacity for localized and sustained release. While traditional hydrogels allow facile drug delivery and possess desirable biocompatibility, inherent limitations such as unsatisfactory loading efficiency and poor release tunability impede therapeutic outcomes in complex lesion microenvironments (Zhou et al., 2026). Hydrogel microspheres, by contrast, offer distinct advantages including high surface-area-to-volume ratio for enhanced drug loading and exchange, superior injectability with minimal invasiveness, modular design flexibility, and deep tissue penetration capability. As demonstrated by recent studies, hydrogel microspheres can serve as versatile platforms for immune modulation and tissue regeneration, achieving therapeutic outcomes that are difficult to attain with bulk hydrogel systems (Li et al., 2023). Gellan gum (GG), an anionic microbial polysaccharide, has been widely explored in tissue engineering and drug delivery owing to its biocompatibility, biodegradability, ion-responsive gelation, and tunable mechanical properties (Liu et al., 2022). In particular, GG-based hydrogels have been investigated as cartilage-related biomaterials because their hydrated network can mimic certain features of the cartilage extracellular matrix (Biswal et al., 2025). γ-Polyglutamic acid (γ-PGA) is another biocompatible and biodegradable polymer with abundant free carboxyl groups, which provide high-density coordination sites and a negatively charged hydrated microenvironment (Gong et al., 2023; Yang et al., 2023). In the present design, GG was used as the primary microsphere-forming framework, while γ-PGA was introduced to enrich carboxylate coordination sites and provide a cartilage-mimetic anionic hydrated network. Upon addition of ZnSO_4_, Zn^2+^ coordinated with carboxylate groups from GG and γ-PGA chains to form a reversible dynamic coordination network, generating PGG-Zn hydrogel microspheres. Notably, metabolic remodeling and enhanced glycolysis in OA can lead to lactate accumulation in the joint microenvironment (Yang et al., 2025; Qian et al., 2026). Because lactate contains both hydroxyl and carboxyl groups and can coordinate with metal ions, elevated lactate may competitively interact with Zn^2+^, weaken Zn^2+^–carboxylate crosslinking points, loosen the microsphere network, and thereby promote drug release (Liu et al., 2025; Tang et al., 2025). OA-associated pH fluctuations trigger on-demand curculigoside release precisely at the lesion site.

Herein, we developed Cur-loaded PGG-Zn hydrogel microspheres (PGG-Zn@Cur) through Zn^2+^-mediated dynamic coordination for local OA intervention. The microspheres exhibited favorable morphology, uniform elemental distribution, coordination-associated chemical features, efficient Cur encapsulation, gradual degradation, and pH-responsive swelling and release behavior. *In vitro*, PGG-Zn@Cur showed good cytocompatibility and protected IL-1β-stimulated chondrocytes by restoring cartilage matrix metabolic homeostasis. Transcriptomic analysis and subsequent validation identified PI3K-AKT signaling and apoptosis-associated responses as candidate mechanisms involved in PGG-Zn@Cur-mediated chondroprotection. *In vivo*, intra-articular administration of PGG-Zn@Cur improved gait symmetry, alleviated mechanical hypersensitivity, preserved proteoglycan-rich cartilage matrix, reduced histological degeneration scores, and attenuated PI3K-AKT phosphorylation and apoptosis-related cartilage damage in OA mice. These findings suggest that PGG-Zn@Cur may serve as a microenvironment-responsive local delivery platform for OA intervention.

## 2 Materials and methods

### 2.1 Synthesis and characterization of PGG-Zn@Cur hydrogel

Low-acyl gellan gum (GG) was dispersed in deionized water and completely dissolved under vigorous stirring in a water bath at 85 °C–90 °C to yield a 2.0% (w/v) aqueous stock solution. Separately, γ-polyglutamic acid (γ-PGA) was added to deionized water and stirred until complete dissolution, yielding a 2.0% (w/v) γ-PGA solution. After the GG solution was cooled to approximately 45 °C–50 °C, it was blended with the γ-PGA solution at a 5:5 ratio (v/v) to achieve final concentrations of 1.0% (w/v) for GG and 1.0% (w/v) for γ-PGA in the polymer system. The mixture was continuously stirred until a homogeneous PGG solution was obtained.

Curculigoside (Cur, HY-N0705, MCE) was initially solubilized in DMSO via ultrasonication to prepare a 100 mM stock solution. This stock solution was subsequently diluted 1:100 (v/v) into the above PGG solution to reach a final Cur concentration of 1 mM. The resulting mixture was thoroughly homogenized in a shaker at 4 °C to afford the PGG/Cur composite solution.

The PGG/Cur mixture was extruded dropwise into a 10 mM ZnSO_4_ crosslinking solution via a syringe pump. Dynamic coordination crosslinking occurred between Zn^2+^ and the carboxylate groups present in both GG and γ-PGA, concurrently entrapping Cur within the hydrogel microsphere network. Following crosslinking for 20 min, the microspheres were collected and thoroughly rinsed to eliminate surface-adsorbed free drug and unbound Zn^2+^, yielding the final PGG-Zn@Cur hydrogel microspheres.

### 2.2 Characterization of PGG-Zn@Cur

The overall morphology and size distribution of PGG-Zn@Cur microspheres were first evaluated using an inverted optical microscope. To further characterize their surface microstructure, the samples were freeze-dried, carefully mounted on conductive carbon tape, followed by morphological observation with a scanning electron microscope (SEM; Regulus 8,100, Hitachi, Japan). Elemental mapping and compositional analysis were performed using energy-dispersive X-ray spectroscopy (EDS) attached to the SEM. In addition, the hydrodynamic particle size of the microspheres was analyzed by dynamic light scattering (DLS).

The degradation profile of PGG-Zn@Cur microspheres was assessed *in vitro* by determining the residual mass of the samples after incubation in phosphate-buffered saline (PBS, pH 7.4). In brief, accurately weighed dry microsphere samples (W_0_) were placed in PBS and maintained at 37 °C with mild agitation. At predefined intervals, the samples were retrieved from the medium and carefully rinsed with deionized water to remove residual surface salts. They were subsequently freeze-dried until no further change in weight was observed. The dried samples were subsequently weighed (W_t_), and the remaining weight percentage was calculated according to the following formula: The *in vitro* degradation behavior of PGG-Zn@Cur was evaluated by monitoring the mass loss over time in phosphate-buffered saline (PBS, pH 7.4). Briefly, pre-weighed dried samples (W_0_) were immersed in PBS and incubated at 37 °C under gentle shaking. At scheduled time points, the samples were taken out and carefully washed with deionized water to eliminate remaining salts on the surface. Afterward, they were freeze-dried until the weight became stable and then reweighed as W_t_. The relative weight (%) was calculated using the following equation:

$$\mathrm{Relative weight}\left(\%\right)=\mathrm{Wt}/W_{0}\times 100\%$$

The Cur entrapment capacity of PGG-Zn@Cur microspheres was evaluated using ultraviolet–visible (UV–Vis) spectrophotometry. The microspheres were separated from the aqueous medium by filtration, and the quantity of free Cur left in the collected liquid phase was analyzed at the maximum absorption wavelength (λmax) of Cur. The total drug content was determined by dissolving a known amount of PGG-Zn@Cur in an organic solvent DMSO to completely disrupt the carrier and release the encapsulated Cur. The EE value was obtained using the following equation:

$$\mathrm{EE}\left(\%\right)=\left(\mathrm{Total drug amount}-\mathrm{Free drug amount}\right)/\mathrm{Total drug amount}\times 100\%$$

The drug loading content (DLC) of Cur in PGG-Zn@Cur microspheres was determined by UV–Vis spectrophotometry. Briefly, a known mass of lyophilized PGG-Zn@Cur microspheres was accurately weighed and incubated in DMSO under sonication to extract the encapsulated Cur from the hydrogel matrix. Following extraction, the mixture was centrifuged to separate undissolved residues, and the clarified liquid phase was retained for UV–Vis measurement. The Cur level was quantified using a standard calibration curve at its characteristic absorption maximum. The DLC value was derived according to the formula below:

$$\mathrm{DLC}\left(\%\right)=W\_\mathrm{Cur}/\;W\_\mathrm{PGG}-\mathrm{Zn}@\mathrm{Cur}\times 100\%$$

Where W_Cur represents the mass of Cur extracted from the PGG-Zn@Cur microspheres, and W_PGG-Zn@Cur microspheres represents the dry weight of the lyophilized Cur-loaded microspheres used for analysis.

The pH-responsive swelling behavior of PGG-Zn@Cur microspheres was evaluated in phosphate-buffered saline (PBS) under neutral physiological conditions (pH 7.4) and acidic OA-like conditions (pH 5.5). Briefly, dry microsphere samples with predetermined weights (W^d^) were incubated in the corresponding buffer solutions at 37 °C. At selected intervals, the samples were removed from the buffer, and the surface-adhered liquid was gently blotted away with filter paper before measuring the swollen mass (Wˢ). The swelling ratio was derived from the equation shown below:

$$\mathrm{Swelling ratio}\left(\%\right)=\left[\left(Wˢ-W^{d}\right)\;/\;W^{d}\right]\times 100\%$$

The release behavior of Cur from PGG-Zn@Cur microspheres was examined under physiological and acidic conditions by incubating the samples in PBS adjusted to pH 7.4 or 5.5. Briefly, a defined quantity of PGG-Zn@Cur microspheres was placed in the release medium and maintained at 37 °C on an orbital shaker. At scheduled sampling points, a fixed volume of medium was collected for UV–Vis analysis, and an equal volume of fresh preheated PBS to keep the release volume constant and preserve sink conditions. The released Cur content was quantified using a standard calibration curve at the characteristic absorption wavelength of Cur. The cumulative release percentage was expressed relative to the total amount of Cur loaded in the microspheres and calculated according to the following equation:

$$Cumulative\;release\left(\%\right)=\frac{V_{e}\sum_{i=1}^{n‐1}C_{i}+V_{0}C_{n}}{m_{\text{drug}}}\times 100\%$$

Where V_0_ represents the initial volume of release medium in the system, V_e_ denotes the sampling volume removed at each time point, C_n_ is the Cur concentration measured at the *n*th sampling point, Cᵢ represents the Cur concentration detected at each previous sampling point, and MCur is the total amount of Cur loaded in the microspheres.

Zeta potential measurements were performed to characterize the surface electrostatic properties of the microspheres. Freeze-dried samples were resuspended in ultrapure water at 0.2 mg/mL and mildly sonicated for 5 min to minimize particle aggregation. After equilibration at 25 °C, the electrophoretic mobility of the dispersed microspheres was determined by electrophoretic light scattering using a Zetasizer instrument equipped with a folded capillary cell. Zeta potential values were calculated according to the Smoluchowski model. Three independent measurements were conducted for each formulation, with at least 10 runs included in each measurement.

### 2.3 Isolation and culture of mouse primary chondrocytes (MPCs)

MPCs were isolated according to a previously reported procedure with minor modifications (Zou et al., 2026). Briefly, after euthanasia, articular cartilage was carefully harvested from the knee joint tissues from C57BL/6 mice aged 2 weeks. The collected cartilage pieces were rinsed several times with sterile phosphate-buffered saline (PBS) and enzymatically digested for 12 h at 37 °C in DMEM/F-12 supplemented with 1 mg/mL collagenase type II (Sigma, C2-BIOC). After digestion, the suspension was passed through a 100 μm nylon cell strainer to eliminate remaining tissue fragments before centrifugation, followed by centrifugation to collect the released cells. The collected cells were subsequently resuspended and seeded in complete DMEM/F-12 containing 10% fetal bovine serum (FBS) and 1% penicillin–streptomycin. To reduce phenotype drift during *in vitro* expansion, passage 3 (P3) cells were used for all downstream experiments.

### 2.4 Cell culture and treatment

MPCs were cultured in complete DMEM/F-12 medium and maintained in a humidified incubator containing 5% CO_2_ at 37 °C. An inflammatory chondrocyte-like condition was induced by exposing the cells to recombinant interleukin-1β (IL-1β, 10 ng/mL; MCE, HY-P7028) for 48 h before further assays. For material-based interventions, blank PGG-Zn microspheres and Cur-loaded PGG-Zn microspheres were separately added to the cell cultures at a final concentration of 50 μg/mL and incubated for 48 h. In the free-drug group, Cur was prepared in complete medium at a final concentration of 1 μg/mL, and the treatment duration was kept consistent with that of the microsphere groups.

### 2.5 *In vitro* biocompatibility evaluation of PGG-Zn@Cur

The cytocompatibility of PGG-Zn@Cur microspheres was assessed by LIVE/DEAD viability staining together with CCK-8 cell proliferation assays. For LIVE/DEAD staining, PGG-Zn@Cur microspheres were preloaded into culture plates, followed by seeding of MPCs at 2 × 10^3^ cells per well. Following cell adherence, the cultures were incubated with PGG-Zn@Cur in the presence of IL-1β (10 ng/mL) for 48 h. The cells were then gently rinsed three times with sterile PBS and stained using the LIVE/DEAD® Viability/Cytotoxicity Kit (C2015S, Beyotime, China) following the supplier’s protocol. The stained cells were visualized and photographed with an inverted fluorescence microscope to assess cell survival.

For the CCK-8 assay, MPCs were dispensed into 96-well plates at 8 × 10^3^ cells per well, followed by a 24-h attachment period before treatment. The cells were subsequently treated with PGG-Zn@Cur, IL-1β (10 ng/mL), or their combination according to the experimental grouping for 24, 48, 72, and 96 h. At each time point, cell viability was determined using a Cell Counting Kit-8 (CCK-8; BS350A, Biosharp, China). Briefly, 10 μL of CCK-8 reagent was added to each well, followed by incubation at 37 °C for 1 h. The optical density was measured at 450 nm with a microplate reader (Thermo Fisher Scientific, USA).

### 2.6 Quantitative real-time polymerase chain reaction (qPCR)

Total RNA was isolated from the collected cells using a SteadyPure RNA Rapid Extraction Kit (Accurate Biotechnology, AG21024) following the recommended procedure. The purified RNA was subsequently converted into first-strand cDNA using Evo M-MLV Reverse Transcription Premix (Accurate Biotechnology, AG11706). RT-qPCR reactions were performed using Taq Pro Universal SYBR qPCR Master Mix (Vazyme, Q712-02) on a Bio-Rad CFX Connect Real-Time PCR System. The relative mRNA abundance of each target gene was normalized against β-Actin and analyzed using the 2^−ΔΔCt approach. The primer sequences applied in this study are provided in Table 1.

**TABLE 1:** qPCR primer information.

Gene name	Primer sequence
*β-Actin FP*	GGC​TGT​ATT​CCC​CTC​CAT​CG
*β-Actin RP*	CCA​GTT​GGT​AAC​AAT​GCC​ATG​T
*Col2a1 FP*	GCT​GCG​GAT​GCT​CTC​AAT​CT
*Col2a1 RP*	TGG​ACG​ATC​AGG​CGA​AAC​C
*Aggrecan FP*	ACA​CTC​AGC​GAG​TTG​TCA​TGG
*Aggrecan RP*	ACT​CTG​GGT​TTT​CGT​GAC​TCT
*Mmp13 FP*	AGG​CTC​CGA​GAA​ATG​CAG​TC
*Mmp13 RP*	CCC​CGC​ATC​TTG​GCT​TTT​TC
*Adamts5 FP*	AAG​TGC​GGA​GTA​TGT​GGA​GGA​G
*Adamts5 RP*	TCT​GGT​CTT​TGG​CTT​TGA​ACT​GTC

### 2.7 Western blot analysis

Protein lysates were prepared from cultured chondrocytes or cartilage tissue samples using RIPA buffer (Beyotime, P0013B) containing phenylmethylsulfonyl fluoride (PMSF) and phosphatase inhibitors. The protein content of each sample was measured with a BCA Protein Assay Kit (Beyotime, P0010). For SDS-PAGE analysis, equal amounts of protein (20–30 μg) were loaded into each lane and separated on 10%–12% polyacrylamide gels, followed by electrotransfer to PVDF membranes. The membranes were blocked with 5% bovine serum albumin (BSA) in TBST for 1 h at room temperature and then incubated overnight at 4 °C with the corresponding primary antibodies. The membranes were subsequently rinsed three times with TBST and further incubated with horseradish peroxidase (HRP)-labeled secondary antibodies at room temperature for 1 h. Immunoreactive bands were developed using an enhanced chemiluminescence (ECL) reagent and recorded with a ChemiDoc Go Imaging System. Densitometric analysis was conducted using ImageJ, and the values were normalized to the corresponding internal control.

The primary antibodies included anti-Aggrecan (ACAN; Santa Cruz, sc-166951), anti-Collagen Type II Alpha 1 Chain (COL2A1; Proteintech, 28459-1-AP), anti-Matrix Metalloproteinase 13 (MMP13; Proteintech, 18165-1-AP), anti-iNOS (Proteintech, 22226-1-AP), anti-PI3K (Proteintech, 67071-1-Ig), anti-phosphorylated PI3K (p-PI3K; HUABIO, HA722951), anti-AKT (Proteintech, 10176-2-AP), anti-phosphorylated AKT (Proteintech, 66444-1-Ig), anti-Caspase-3 (Proteintech, 19677-1-AP), anti-Cleaved Caspase-3 (Cl-Caspase-3; Proteintech, 68773-1-Ig), anti-Bax (Proteintech, 50599-2-Ig), anti-BCL2 (Proteintech, 60178-1-Ig), and anti-β-Actin (Proteintech, 10201-2-AP).

### 2.8 Immunofluorescence (IF) staining

For immunofluorescence analysis, MPCs were seeded onto 12-mm glass coverslips placed in 24-well plates and cultured until attachment. Cells grown on coverslips were fixed with 4% paraformaldehyde (PFA) at room temperature for 15 min, followed by washing three times with PBS. Cellular membranes were permeabilized using 0.3% Triton X-100 prepared in PBS for 10 min. After washing, nonspecific binding sites were blocked with 1% bovine serum albumin (BSA) in PBS at room temperature about 1 h. Primary antibody staining was performed by maintaining the samples in the corresponding antibody solutions overnight at 4 °C targeting COL2A1, MMP13, p-PI3K, p-AKT, or Cleaved Caspase-3 at a dilution of 1:200. After three PBS washes, the coverslips were exposed to the appropriate fluorescent secondary antibody, including Alexa Fluor® 488-conjugated goat anti-rabbit IgG (H + L) (Beyotime, A0423) or Alexa Fluor® 555-conjugated donkey anti-rabbit IgG (H + L) (Beyotime, A0453), at a dilution of 1:500 at 37 °C for about 1 h protected from light. Nuclei were labeled with DAPI (1:5,000; Sigma-Aldrich, D8417) for 5 min. Following the final PBS rinses, the coverslips were sealed with antifade mounting medium, and fluorescence images were obtained using a Fluoview FV4000 laser scanning confocal microscope (Olympus, Japan).

### 2.9 Animal models

Male C57BL/6J mice were procured from Chongqing Enshiwei Biotechnology Co., Ltd. (license No. SCXK [Xiang] 2021–0002). The animals were subsequently housed under specific pathogen-free (SPF) conditions at the Laboratory Animal Center of the Second Affiliated Hospital, Chongqing Medical University. All procedures involving animals were conducted in accordance with the ARRIVE guidelines and approved by the Animal Ethics Committee of the Second Affiliated Hospital of Chongqing Medical University (Approval No.: IACUC-SAHCQMU-2026–0107). Osteoarthritis was established in 8-week-old male C57BL/6 mice using the destabilization of the medial meniscus (DMM) model. Mice were anesthetized by intraperitoneal injection of sodium pentobarbital at a dose of 50 mg/kg before surgery, the right knee was prepared for surgery. A medial parapatellar incision was created, and the subcutaneous tissue together with the joint capsule was carefully separated to visualize the medial meniscotibial ligament (MMTL). The MMTL was sharply divided with a microsurgical blade, thereby inducing medial meniscal instability. The joint capsule and skin were then closed layer by layer using absorbable sutures for the capsule and 6–0 silk sutures for the skin. In sham-operated mice, the right knee joint was exposed through the same approach, but the MMTL was left intact. After surgery, mice were housed individually under standard conditions with unrestricted cage activity. Buprenorphine was given subcutaneously at 0.1 mg/kg every 12 h for the first 48 h to provide postoperative analgesia.

For intra-articular treatment, PGG-Zn@Cur or blank PGG-Zn microspheres were delivered into the right knee joint through a medial parapatellar injection approach beginning 1 week after surgery. Before administration, the microspheres were dispersed in sterile PBS at 5 mg/mL and gently vortexed to obtain a homogeneous suspension. Each mouse received 20 μL of the corresponding formulation once per week. Briefly, after disinfection of the knee skin with 75% ethanol, intra-articular access was achieved by inserting a 26-gauge needle attached to a 1-mL syringe through the medial aspect of the patellar ligament. Mice in the sham-operated and DMM-control groups were injected with an equivalent volume of sterile saline according to the identical schedule and injection route. For the free-drug group, Cur was prepared in sterile PBS at 75 μg/mL and administered intra-articularly using the same frequency and dosing regimen described above.

### 2.10 Anesthesia and euthanasia

Prior to surgery and euthanasia, mice were deeply anesthetized via intraperitoneal injection of pentobarbital sodium (Sigma-Aldrich, P3761; dissolved in sterile water to a concentration of 1% w/v). The anesthetic dose was adjusted according to individual body weight at 50 mg/kg. The depth of anesthesia was verified by the absence of toe-pinch withdrawal reflex and loss of corneal reflex before proceeding.

Mice were humanely euthanized prior to tissue collection, with procedures detailed below. Following confirmed deep anesthesia, mice were euthanized by CO_2_ inhalation using a calibrated CO_2_ euthanasia chamber (SMQ-II, Minlylab, China). Compressed CO_2_ was delivered at a displacement rate of 30% of the chamber volume per minute, gradually raising the CO_2_ concentration to ≥70%. The 70% CO_2_ atmosphere was maintained for at least 5 min. Cervical dislocation was subsequently performed as a physical secondary method to ensure death. Death was confirmed by the cessation of respiration, absence of heartbeat, loss of corneal reflex, and onset of rigor mortis. No tissue collection was performed until all criteria were satisfied.

### 2.11 Mouse gait analysis

The CatWalk XT gait analysis system (Noldus Information Technology, Wageningen, Netherlands) was calibrated according to the manufacturer’s protocol prior to testing. Before behavioral assessment, mice were allowed to adapt to the experimental environment and apparatus for three consecutive days (10 min per day) to minimize stress-induced locomotor variability. For data acquisition, each mouse was placed individually in the illuminated runway chamber and allowed to traverse the glass plate at a constant speed without interruption. Gait parameters—including stance duration, stride length, mean intensity, and print area—were automatically recorded by the integrated high-speed video capture system. Only runs meeting the quality criteria (continuous locomotion with a speed variation coefficient <60% and a minimum of five complete step cycles) were retained for analysis. Each animal underwent three valid consecutive runs per session. Raw data were processed and quantified using CatWalk XT 10.5 software (Noldus Information Technology). All mice were subjected to gait analysis 24 h prior to euthanasia at 8 weeks post-surgery.

### 2.12 Open field test

Open-field testing was performed to assess the spontaneous movement behavior of mice measuring 50 × 50 × 40 cm. Under an illumination intensity of 40–60 lux, each mouse was gently introduced into the central area of the arena and monitored for 5 min without external interference. Behavioral parameters, including total movement distance, duration in the center area, and the number of vertical rearings, were automatically tracked and processed using EthoVision XT 14 software (Noldus Information Technology). To avoid odor-related interference, the floor of the apparatus was cleaned with 70% ethanol after each trial and fully dried before testing the next animal. All recordings were performed in a temperature-controlled room during the light cycle between 9:00 and 15:00. Open-field testing was conducted 24 h before tissue harvesting at 8 weeks after surgery.

### 2.13 Von frey filament test

Mechanical sensitivity was assessed with the Von Frey up-down paradigm. Before testing, mice were placed separately on a raised wire-mesh platform and habituated to the apparatus for 1 h. Calibrated Von Frey filaments ranging from 0.1 to 20 g were gently applied to the plantar region of the hind paw at a right angle for 1–2 s. Measurements were first performed on the left hind paw, which was contralateral to the operated knee, and then on the right hind paw, which corresponded to the DMM-operated side. A rapid withdrawal of the paw or licking after stimulation was recorded as a positive reaction. According to the up-down testing sequence, the next stimulus was increased after no response and decreased after a positive response. After the initial response reversal, each paw received five additional stimulations, with intervals of at least 2 min between applications to reduce repeated-stimulation sensitization. Dixon’s up-down calculation was applied to determine the 50% paw withdrawal threshold. Behavioral measurements were repeated once weekly from postoperative week 1 to week 8.

### 2.14 Safranin O/Fast green staining

Harvested cartilage specimens and mouse knee joint specimens were fixed with 4% paraformaldehyde (PFA) at 4 °C for 72 h before histological preparation. After fixation, cartilage explants were processed through serial ethanol dehydration and directly embedded in paraffin. For mouse knee samples, decalcification was carried out in 15% EDTA solution (pH 7.4) at 37 °C for 2 weeks, followed by routine paraffin embedding. The embedded samples were cut into 5-μm-thick sections using a Minux® S700A rotary microtome. To improve section adherence, the paraffin-embedded slides were baked at 60 °C for 2 h, followed by xylene deparaffinization and stepwise rehydration through decreasing concentrations of ethanol to deionized water. Modified Safranin O/Fast Green staining was then conducted according to the staining protocol. Briefly, tissue sections were processed through successive staining steps with hematoxylin, Fast Green, and Safranin O for 5, 10, and 5 min, respectively, with distilled water rinsing between staining steps. Histological cartilage damage in murine knee joints was evaluated according to the OARSI grading criteria and the modified Mankin scoring method (Pritzker et al., 2006; Glasson et al., 2010). All histological images were anonymized before evaluation and independently assessed by two observers blinded to the experimental groups. For each sample, the final histological score was calculated as the average value of the two independent assessments.

### 2.15 Paraffin section IF staining

Paraffin-embedded tissue sections were processed for antigen exposure by trypsin-mediated retrieval at 37 °C for 30 min. After retrieval, nonspecific binding was minimized by incubation using normal goat serum for 30 min at 37 °C. The sections were subsequently reacted with primary antibodies targeting COL2A1 (Proteintech, 28459-1-AP), MMP13 (Proteintech, 18165-1-AP), p-PI3K (HUABIO, HA721672), and p-AKT (Proteintech, 66444-1-Ig) for 16 h at 4 °C. Following rinsing with TBST, the sections were incubated in the dark with Alexa Fluor® 555-conjugated donkey anti-rabbit IgG (1:400 in TBST) at room temperature for about 1 h. After staining, the sections were mounted with antifade medium containing DAPI, and fluorescence signals were visualized using a Fluoview FV4000 laser scanning confocal microscope (Olympus, Japan).

### 2.16 TUNEL staining

Apoptotic cells in paraffin-embedded sections were detected using a TUNEL assay kit (Beyotime, C1089). Before labeling, tissue sections underwent treatment with trypsin at 37 °C for 30 min to improve reagent penetration, and nonspecific background was reduced by incubation with normal goat serum for 30 min at 37 °C. The sections were then exposed to the TUNEL working solution, prepared by mixing enzyme solution and labeling solution at 1:9, and subjected to 60 min in a light-protected humid chamber at 37 °C. After completion of the labeling reaction, nuclei were subsequently performed using DAPI (1 μg/mL) for 5 min at room temperature. Following the final rinses, the slides were sealed with antifade mounting medium, and fluorescence signals were recorded using a Fluoview FV4000 laser scanning confocal microscope (Olympus, Japan).

### 2.17 RNA sequencing

Cellular total RNA was extracted using TRIzol reagent from primary murine chondrocytes subjected to IL-1β stimulation with or without PGG-Zn@Cur treatment, with three biological replicates included in each group. All extraction procedures were carried out following the protocol that the supplier recommended. RNA quality was examined by agarose gel electrophoresis, while RNA yield and purity were assessed with a NanoDrop spectrophotometer, with samples showing an A260/A280 ratio of at least 1.8 used for subsequent analysis. Library preparation, RNA sequencing, and initial quality control were completed by Majorbio Bio-Pharm Technology Co., Ltd. (Shanghai, China). Briefly, polyadenylated mRNA was captured using oligo (dT)-coated magnetic beads, fragmented, and then converted into cDNA by reverse transcription. Sequencing libraries were constructed using the TruSeq RNA Sample Preparation Kit (Illumina) and analyzed on an Illumina NovaSeq 6,000 platform to obtain 150-bp paired-end reads. DESeq2 software (version 1.34.0) was applied to screen genes showing differential expression. Significant differential expression was determined using cutoff criteria of adjusted p value ≤0.05 and |log_2_ fold change| ≥ 1. Gene Ontology (GO) functional enrichment was analyzed with the clusterProfiler package (version 4.2.2), and a corrected p value <0.05 was used as the threshold for significant GO enrichment. Data supporting the findings of this work can be obtained from the corresponding author on reasonable request.

### 2.18 Statistical analysis

Quantitative results are expressed as the mean ± standard error of the mean (SEM). Data processing and statistical testing were carried out with GraphPad Prism 9.0 software (GraphPad Software, San Diego, CA, USA). Unless specified otherwise in the corresponding figure legends, comparisons involving two unrelated groups were analyzed using a two-tailed unpaired Student’s t-test, whereas datasets containing three or more groups were evaluated by one-way ANOVA with *post hoc* correction for multiple comparisons. CCK-8 data obtained across different treatment groups and time points were evaluated by two-way ANOVA. Each experiment was repeated independently no fewer than three times. Statistical significance was set at p < 0.05. Significance levels were indicated as follows: ****p < 0.0001, ***p < 0.001, **p < 0.01, and *p < 0.05; ns represents no statistically significant difference (p ≥ 0.05).

## 3 Results

### 3.1 Preparation and characterization of PGG-Zn@Cur hydrogel microspheres

To achieve intra-articular delivery of Curculigoside (Cur) with pH-responsive release for osteoarthritis therapy, PGG-Zn@Cur hydrogel microspheres were fabricated via a dynamic Zn^2+^-carboxylate coordination crosslinking strategy. As illustrated in Figure 1A, low-acyl gellan gum (GG) and γ-polyglutamic acid (γ-PGA) were first dissolved to form a precursor solution, into which Cur was subsequently incorporated. The mixture was then extruded dropwise into a ZnSO_4_ bath, where Zn^2+^ ions coordinated with the carboxylate groups of both GG and γ-PGA to form hydrogel microspheres, concurrently entrapping Cur within the three-dimensional network.

**FIGURE 1:**
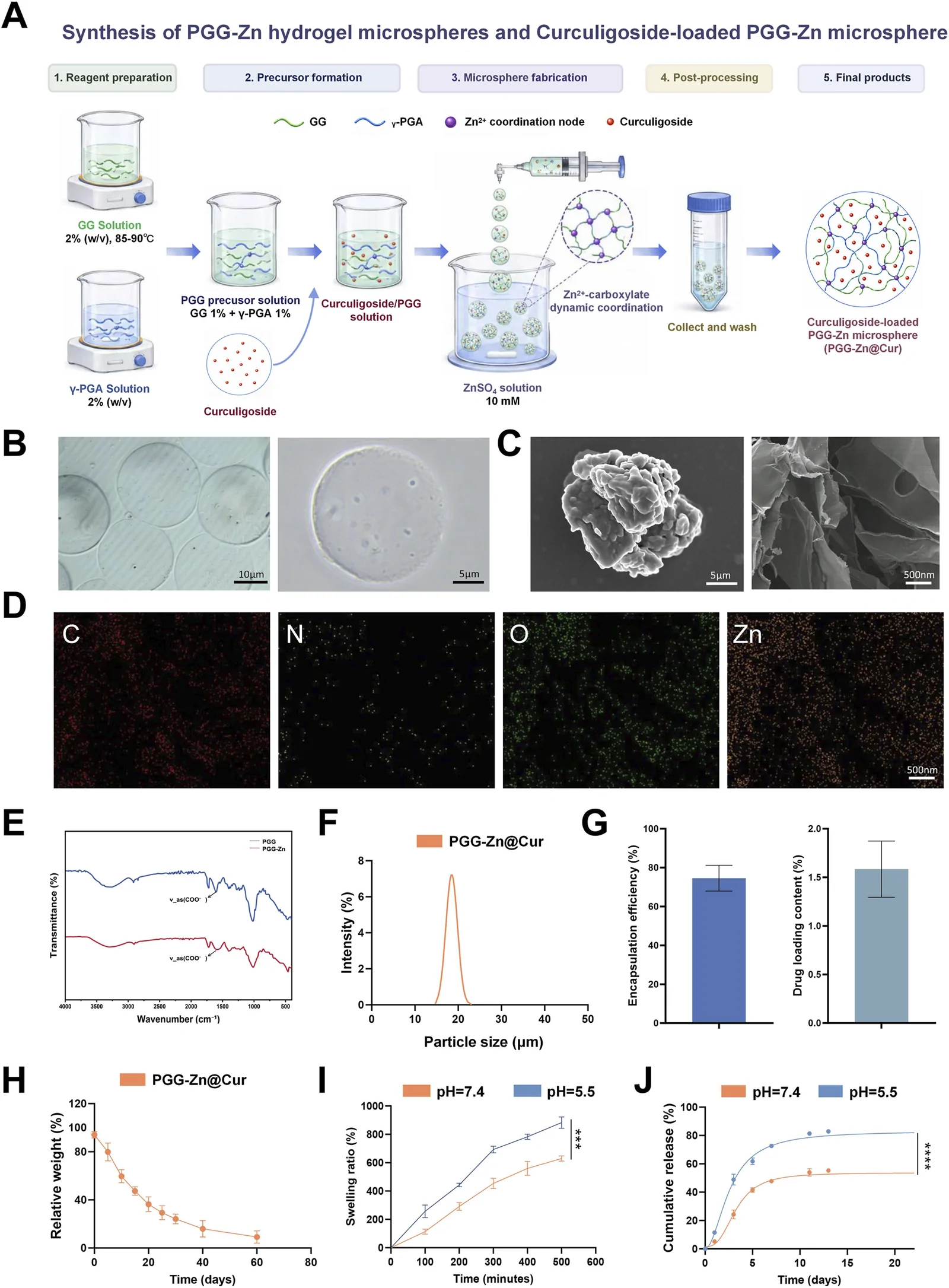
Preparation and characterization of PGG-Zn@Cur hydrogel microspheres. **(A)** Schematic illustration of the preparation of PGG-Zn@Cur hydrogel microspheres. **(B)** Optical microscopy images showing the spherical morphology and uniform size distribution of PGG-Zn@Cur microspheres. **(C)** Scanning electron microscopy (SEM) images of lyophilized PGG-Zn@Cur microspheres. **(D)** Energy-dispersive X-ray spectroscopy (EDS) elemental mapping demonstrating the homogeneous distribution of carbon **(C)**, nitrogen (N), oxygen (O), and zinc (Zn) within the microspheres. **(E)** Fourier-transform infrared (FTIR) spectra of PGG and PGG-Zn, showing the characteristic shift of the carboxylate asymmetric stretching vibration (ν_as (COO^−^)) upon Zn^2+^ coordination. **(F)** Dynamic light scattering (DLS) analysis of PGG-Zn@Cur microspheres. **(G)** Encapsulation efficiency and drug loading content of Cur in PGG-Zn@Cur microspheres. **(H)** *In vitro* degradation profile of PGG-Zn@Cur microspheres in PBS at 37 °C. **(I)** pH-responsive swelling behavior of PGG-Zn@Cur microspheres in PBS at pH 7.4 and pH 5.5 at 37 °C. **(J)** *In vitro* cumulative release profiles of Cur from PGG-Zn@Cur microspheres at pH 7.4 and pH 5.5. (*n* = 3, *: *p* < 0.05; **: *p* < 0.01; ***: *p* < 0.001; ****: *p* < 0.0001; ns: no significance).

The structural features of the prepared microspheres were initially characterized using optical microscopy and SEM. Optical images showed that PGG-Zn@Cur formed well-defined spherical particles with a relatively homogeneous size distribution and favorable dispersibility (Figure 1B). SEM images further confirmed the spherical structure and revealed a rough, porous surface topography at higher magnification (Figure 1C), a feature favorable for efficient drug loading and diffusion. Energy-dispersive X-ray spectroscopy (EDS) elemental mapping demonstrated the homogeneous distribution of C, N, O, and Zn throughout the microspheres (Figure 1D), confirming the successful incorporation of Zn^2+^ into the hydrogel network.

Fourier-transform infrared (FTIR) spectroscopy was employed to verify the chemical crosslinking mechanism. As shown in Figure 1E, compared with the pristine PGG precursor, the PGG-Zn spectrum exhibited a characteristic shift in the asymmetric stretching vibration of carboxylate groups (ν_as (COO^−^)), indicating the formation of dynamic coordination bonds between Zn^2+^ and the carboxylate moieties of GG/γ-PGA. Dynamic light scattering (DLS) analysis showed the particle size distribution with a mean diameter of approximately 20 μm (Figure 1F). These results demonstrate that PGG-Zn@Cur could exerts therapeutic effects through pH-responsive extracellular release.

The drug encapsulation efficiency of the microspheres was subsequently quantified. The encapsulation efficiency of Cur in PGG-Zn@Cur microspheres reached approximately 70% (Figure 1G). The drug loading content (DLC) was calculated to be approximately 1.5% (Figure 1G), indicating efficient incorporation of Cur into the PGG-Zn hydrogel matrix. Furthermore, the degradation profile demonstrated that the PGG-Zn@Cur microspheres underwent gradual hydrolytic degradation, retaining approximately 10% of their initial dry weight by day 60 (Figure 1H), which supports their potential for long-term intra-articular retention and sustained drug release.

Given the mildly acidic microenvironment characteristic of osteoarthritic joints, the pH-responsive swelling and drug release behaviors were evaluated. The microspheres exhibited significantly higher swelling ratios at pH 5.5 compared to pH 7.4, reaching approximately 800% versus 600% at equilibrium, respectively (Figure 1I). This pH-dependent swelling is attributed to the protonation-mediated dissociation of dynamic Zn^2+^-carboxylate coordination bonds under acidic conditions, which reduces crosslinking density and facilitates network expansion. Correspondingly, *in vitro* release studies revealed a markedly accelerated Cur release profile at pH 5.5, with cumulative release reaching approximately 80% by day 20, compared to only 55% at pH 7.4 (Figure 1J). Additionally, the zeta potential of PGG-Zn@Cur was characterized across a range of pH conditions. The zeta potential became progressively more negative as the pH increased, changing from approximately −19.5 mV at pH 5.5 to −25.0 mV at pH 7.4 and −28.5 mV at pH 9.5 (Supplementary Figure S1A). This trend was attributed primarily to the increased deprotonation of carboxyl groups within the GG/γ-PGA network under neutral and alkaline conditions. Conversely, partial protonation under acidic conditions reduced the surface negative charge. Collectively, these results demonstrate that the PGG-Zn@Cur hydrogel microspheres possess uniform morphology, quantifiable drug loading capacity, sustained degradability, and pronounced pH-responsive swelling and release properties, rendering them promising candidates for targeted OA therapy.

### 3.2 Biocompatibility of PGG-Zn@Cur

We first evaluated whether the material system itself induced overt cytotoxicity in chondrocytes. Chondrocytes were treated with Control, IL-1β, IL-1β + PGG-Zn, IL-1β + Cur, or IL-1β + PGG-Zn@Cur, followed by Live/Dead staining and CCK-8 assays to assess cell viability and metabolic activity. LIVE/DEAD staining was performed to visually assess chondrocyte survival following various treatments. Intense green fluorescence, indicative of live cells, was predominantly observed across all groups, with only sporadic red fluorescent signals corresponding to dead cells. This suggests that primary murine chondrocytes maintained robust viability under all tested conditions. Importantly, treatment with blank PGG-Zn microspheres, free Cur, or PGG-Zn@Cur did not induce significant additional cytotoxicity (Figure 2A). Quantitative analysis further revealed comparable cell viability across all treatment groups, indicating that both the unloaded PGG-Zn vehicle and the Cur-loaded PGG-Zn@Cur system exhibit excellent cytocompatibility (Figure 2B).

**FIGURE 2:**
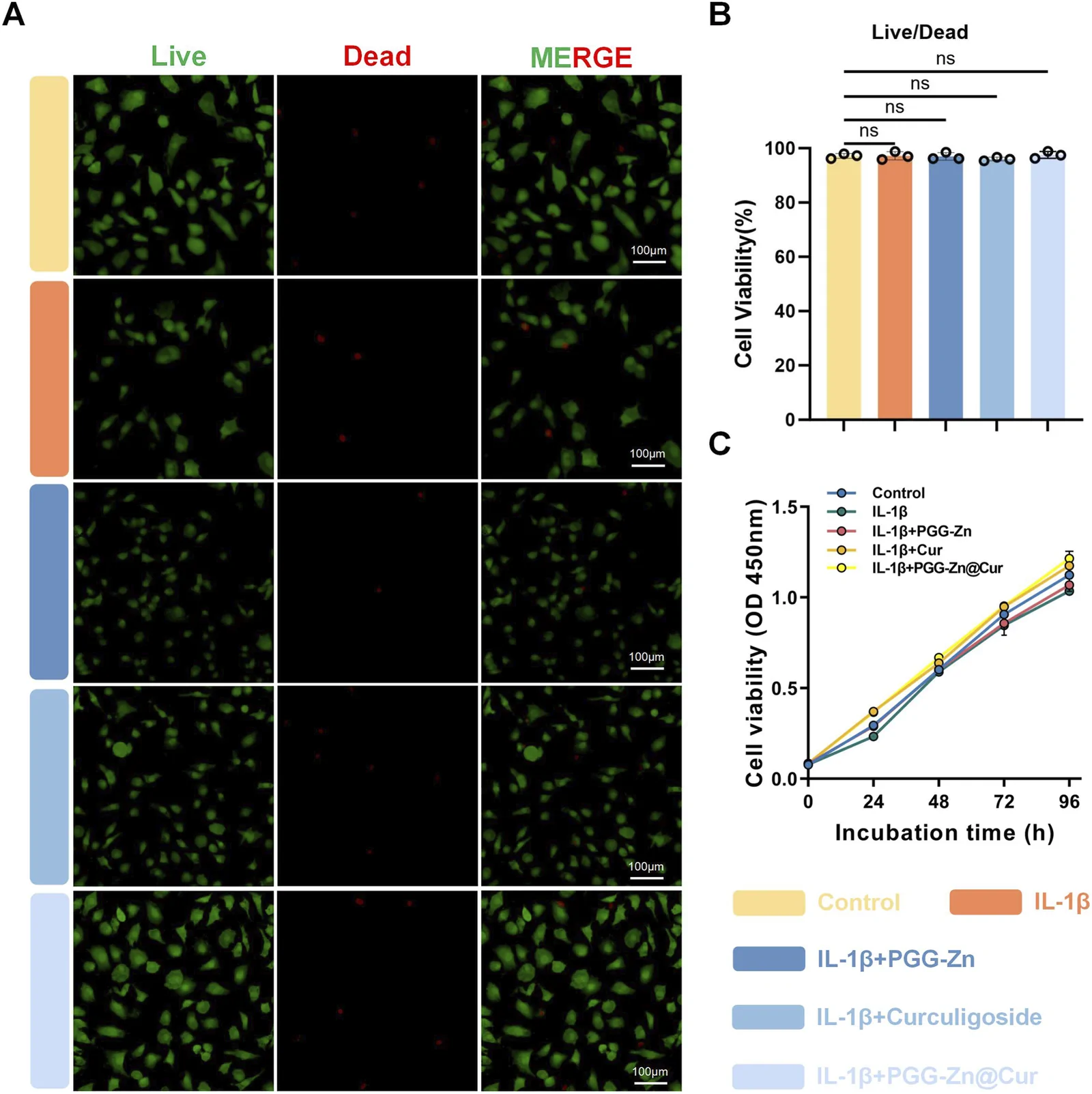
Biocompatibility of PGG-Zn@Cur. **(A)** Representative fluorescence images of Live/Dead staining in chondrocytes after different treatments. Live cells were stained green with calcein-AM, whereas dead cells were stained red with PI. **(B)** Quantitative analysis of cell viability based on Live/Dead staining. No significant difference was observed among the indicated groups, suggesting that PGG-Zn and PGG-Zn@Cur did not induce obvious cytotoxicity in chondrocytes. **(C)** CCK-8 assay showing chondrocyte viability at 0, 24, 48, 72, and 96 h after different treatments. Statistical significance was not annotated in the line graph for clarity. (*n* = 3; Scale bar: 100 μm; *p < 0.05; **p < 0.01; ***p < 0.001; ****p < 0.0001; ns, not significant).

We next examined the metabolic activity of chondrocytes during prolonged incubation using the CCK-8 assay. From 0 to 96 h, the optical density values gradually increased in all groups, suggesting that chondrocytes maintained continuous metabolic activity and growth during the observation period. Compared with IL-1β stimulation alone, PGG-Zn@Cur did not suppress chondrocyte viability at any tested time point and showed a tendency to preserve cell metabolic activity during later culture stages (Figure 2C). Notably, the blank PGG-Zn carrier also exhibited no apparent inhibitory effect on cell growth, indicating that the material matrix possessed acceptable cellular compatibility. These results demonstrate that PGG-Zn@Cur maintains favorable cytocompatibility with chondrocytes.

### 3.3 PGG-Zn@Cur restored matrix metabolic homeostasis in IL-1β-stimulated chondrocytes

To determine whether PGG-Zn@Cur could alleviate inflammatory microenvironment–induced cartilage matrix metabolic imbalance, an OA-like cellular model was established by IL-1β stimulation of primary murine chondrocytes, followed by treatment with PGG-Zn, free Cur, or PGG-Zn@Cur. Immunofluorescence (IF) staining revealed abundant COL2A1 fluorescence in the control group, which was markedly diminished upon IL-1β stimulation. Notably, PGG-Zn@Cur treatment restored COL2A1 fluorescence intensity to a greater extent than either PGG-Zn or Cur alone (Figure 3A). Concurrently, IL-1β stimulation induced pronounced MMP13 accumulation, indicative of enhanced catabolic activation; this effect was substantially attenuated following PGG-Zn@Cur treatment, as evidenced by reduced MMP13-positive fluorescent signals (Figure 3B). Quantitative fluorescence analysis further confirmed that PGG-Zn@Cur significantly preserved COL2A1 expression and suppressed IL-1β–induced MMP13 upregulation (Figure 3C).

**FIGURE 3:**
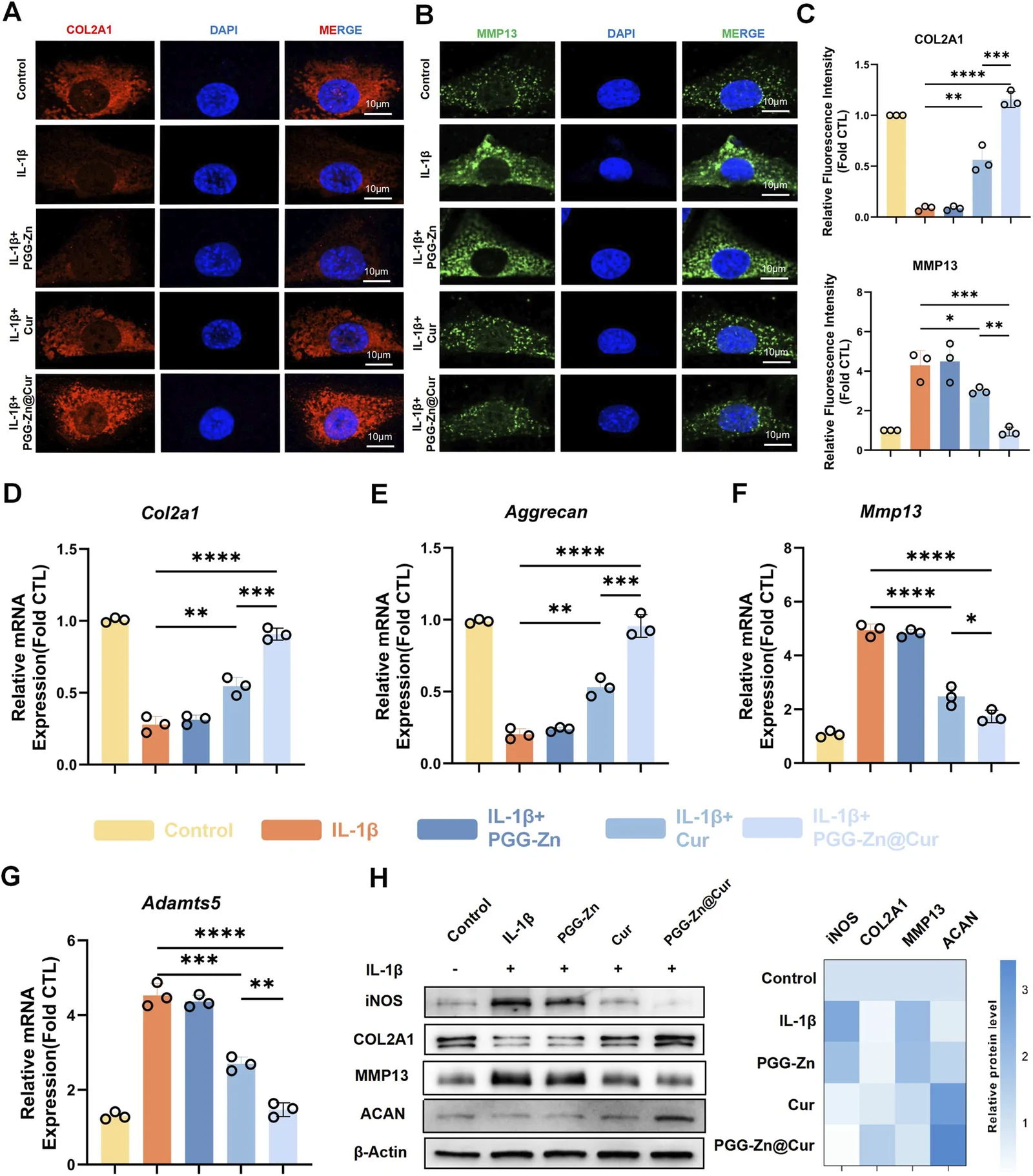
PGG-Zn@Cur restored matrix metabolic homeostasis in IL-1β-stimulated chondrocytes. **(A)** Representative immunofluorescence images of COL2A1 in chondrocytes after different treatments. COL2A1 was stained red, and nuclei were counterstained with DAPI. **(B)** Representative immunofluorescence images of MMP13 in chondrocytes after different treatments. MMP13 was stained green, and nuclei were counterstained with DAPI. **(C)** Quantitative analysis of relative fluorescence intensity for COL2A1 and MMP13 based on immunofluorescence staining. IL-1β stimulation decreased COL2A1 expression and increased MMP13 accumulation, whereas PGG-Zn@Cur treatment restored COL2A1 fluorescence intensity and suppressed MMP13-positive signals. **(D–G)** RT-qPCR analysis of cartilage matrix metabolism-related genes, including anabolic markers Col2a1 and Aggrecan, and catabolic markers Mmp13 and Adamts5, in chondrocytes after the indicated treatments. **(H)** Representative Western blot images of iNOS, COL2A1, MMP13, and ACAN protein expression, together with heatmap-based quantitative analysis of Western blot results across different treatment groups. β-Actin was used as the loading control. (*n* = 3, Scale bar: 10 μm *: *p* < 0.05; **: *p* < 0.01; ***: *p* < 0.001; ****: *p* < 0.0001; ns: no significant).

We further assessed the influence of PGG-Zn@Cur on the expression of genes involved in cartilage matrix homeostasis. RT-qPCR analysis showed that IL-1β exposure decreased the mRNA levels of the anabolic markers Col2a1 and Aggrecan, whereas the catabolic markers Mmp13 and Adamts5 were notably increased. In contrast, treatment with PGG-Zn@Cur partially reversed these IL-1β-induced changes, as evidenced by elevated Col2a1 and Aggrecan expression and reduced Mmp13 and Adamts5 levels compared with the IL-1β group (Figures 3D–G). These results indicate that PGG-Zn@Cur helps preserve matrix metabolic balance in inflammatory chondrocytes by attenuating the IL-1β-driven shift toward a catabolic state.

Western blot analysis further revealed that IL-1β stimulation increased the expression of inflammatory and catabolic proteins iNOS and MMP13, concomitant with decreased levels of COL2A1 and ACAN. PGG-Zn@Cur treatment reversed these expression patterns, as manifested by reduced iNOS and MMP13 levels alongside restored COL2A1 and ACAN expression (Figure 3H). Collectively, these results demonstrate that PGG-Zn@Cur effectively attenuates IL-1β–induced inflammatory responses and catabolic activation in chondrocytes, while preserving the expression of cartilage matrix synthesis–associated markers.

### 3.4 PGG-Zn@Cur attenuated PI3K-AKT activation and apoptosis-associated responses in IL-1β-stimulated chondrocytes

To explore the molecular changes associated with the chondroprotective activity of PGG-Zn@Cur, RNA sequencing was carried out in IL-1β-stimulated chondrocytes with or without PGG-Zn@Cur intervention (Figure 4A). Heatmap clustering and volcano plot visualization showed that PGG-Zn@Cur markedly altered the expression pattern of genes related to cartilage matrix turnover, inflammatory signaling, apoptosis-associated processes, and PI3K-AKT pathway activity (Figures 4B,C). Functional annotation of the identified differentially expressed genes was then performed using Gene Ontology (GO) and Kyoto Encyclopedia of Genes and Genomes (KEGG) analyses. GO enrichment results indicated that these genes were mainly involved in extracellular matrix organization, regulation of inflammatory responses, cell death or apoptotic processes, and kinase-associated signaling events (Figure 4D). KEGG pathway analysis further highlighted PI3K-AKT signaling as a potentially important pathway associated with the biological effects of PGG-Zn@Cur in inflammatory chondrocytes (Figure 4E).

**FIGURE 4:**
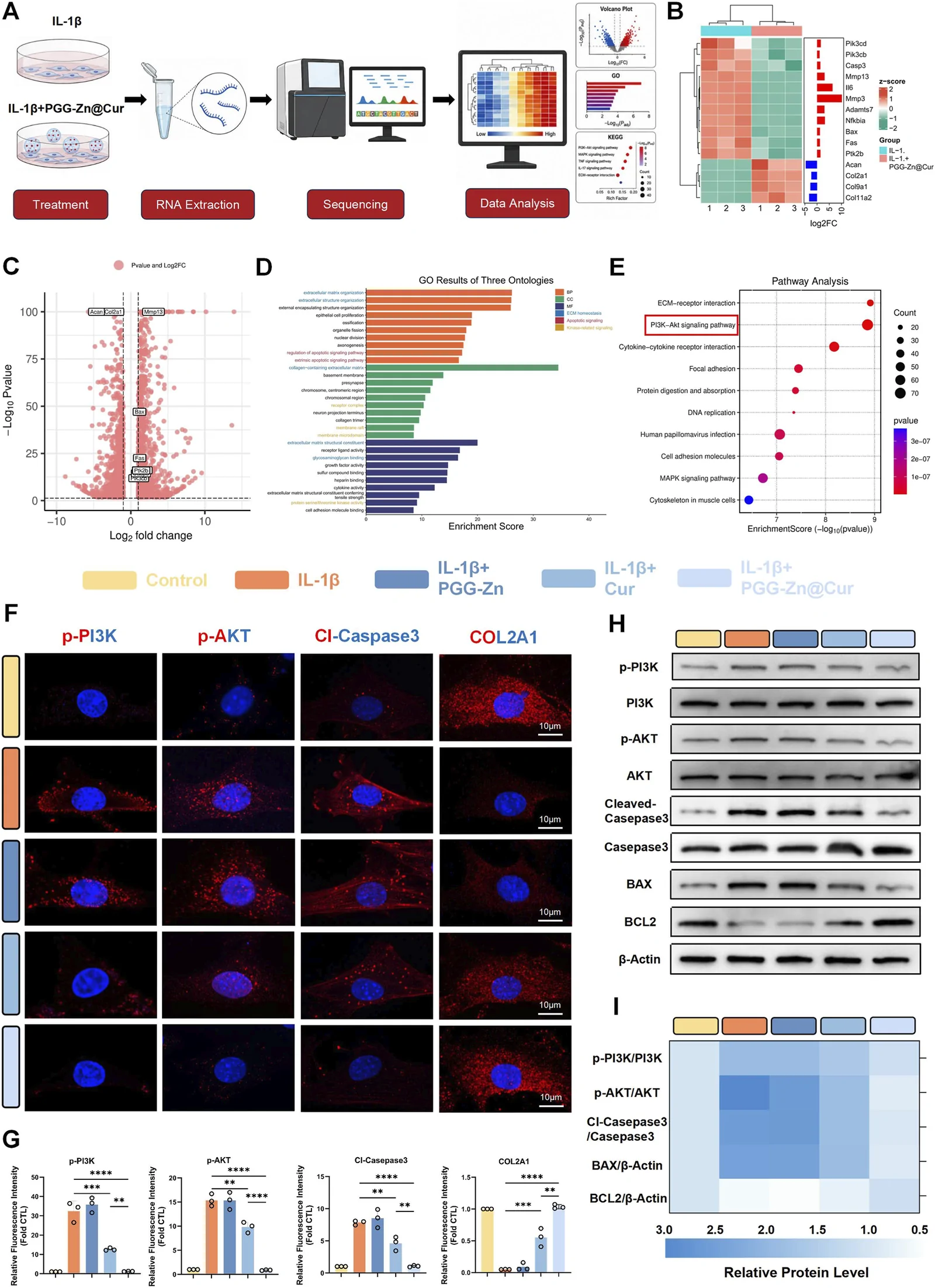
PGG-Zn@Cur attenuated PI3K-AKT activation and apoptosis-associated responses in IL-1β-stimulated chondrocytes. **(A)** Schematic workflow of RNA sequencing and subsequent experimental validation. **(B)** Heatmap of representative differentially expressed genes related to cartilage matrix metabolism, inflammation, apoptosis, and PI3K-AKT-associated signaling. **(C)** Volcano plot showing differentially expressed genes between the indicated groups, with representative genes related to cartilage homeostasis, apoptosis, and PI3K-AKT signaling labeled. **(D)** GO enrichment analysis of differentially expressed genes, including biological process, cellular component, and molecular function categories. **(E)** KEGG pathway enrichment analysis, with the PI3K-AKT signaling pathway highlighted as a candidate pathway. **(F)** Representative immunofluorescence images of p-PI3K, p-AKT, Cl-Caspase3, and COL2A1 in chondrocytes after different treatments. Nuclei were counterstained with DAPI. **(G)** Quantitative analysis of p-PI3K, p-AKT, Cl-Caspase3, and COL2A1 fluorescence intensity. **(H)** Representative Western blot images of PI3K-AKT pathway- and apoptosis-related proteins, including p-PI3K, PI3K, p-AKT, AKT, Cleaved-Caspase3, Caspase3, Bax, and BCL2. β-Actin was used as the loading control. **(I)** Heatmap-based quantitative analysis of Western blot results, summarizing changes in p-PI3K/PI3K, p-AKT/AKT, Cleaved-Caspase3/Caspase3, Bax/β-Actin, and BCL2/β-Actin across different groups. (*n* = 3, Scale bar: 10 μm *: *p* < 0.05; **: *p* < 0.01; ***: *p* < 0.001; ****: *p* < 0.0001; ns: not significant).

To experimentally validate whether PGG-Zn@Cur exerts its protective effects associated with the PI3K-AKT pathway, IF staining and Western blot analyses were performed. IF revealed that PGG-Zn@Cur treatment markedly attenuated IL-1β–induced activation of p-PI3K, p-AKT, and Cl-Caspase-3, while concurrently restoring COL2A1 fluorescence intensity. These findings suggest that PGG-Zn@Cur alleviates PI3K-AKT hyperactivation and apoptosis-associated damage, thereby preserving the cartilage matrix phenotype (Figures 4F,G). Consistent with the immunofluorescence observations, Western blotting further confirmed that IL-1β exposure enhanced PI3K and AKT phosphorylation, accompanied by increased expression of the apoptosis-related markers Cleaved Caspase-3 and Bax and a reduction in BCL2. In contrast, PGG-Zn@Cur intervention markedly lowered the relative phosphorylation ratios of PI3K and AKT, suppressed Cleaved Caspase-3 and Bax expression, and partially recovered BCL2 abundance (Figures 4H,I). Collectively, these results demonstrate that PGG-Zn@Cur protects chondrocytes from IL-1β–induced injury associated with PI3K-AKT signaling and downstream apoptotic cascades.

### 3.5 PGG-Zn@Cur improved gait and alleviated pain-related behavioral dysfunction in OA mice

To evaluate the *in vivo* functionality of PGG-Zn@Cur, an OA mouse model was established via destabilization of the medial meniscus (DMM) in C57BL/6 mice, followed by intra-articular injection of the therapeutic agents. Prior to histological assessment, behavioral evaluations were sequentially performed, including CatWalk gait analysis, Von Frey mechanical allodynia testing, and open field exploration assays (Figure 5A).

**FIGURE 5:**
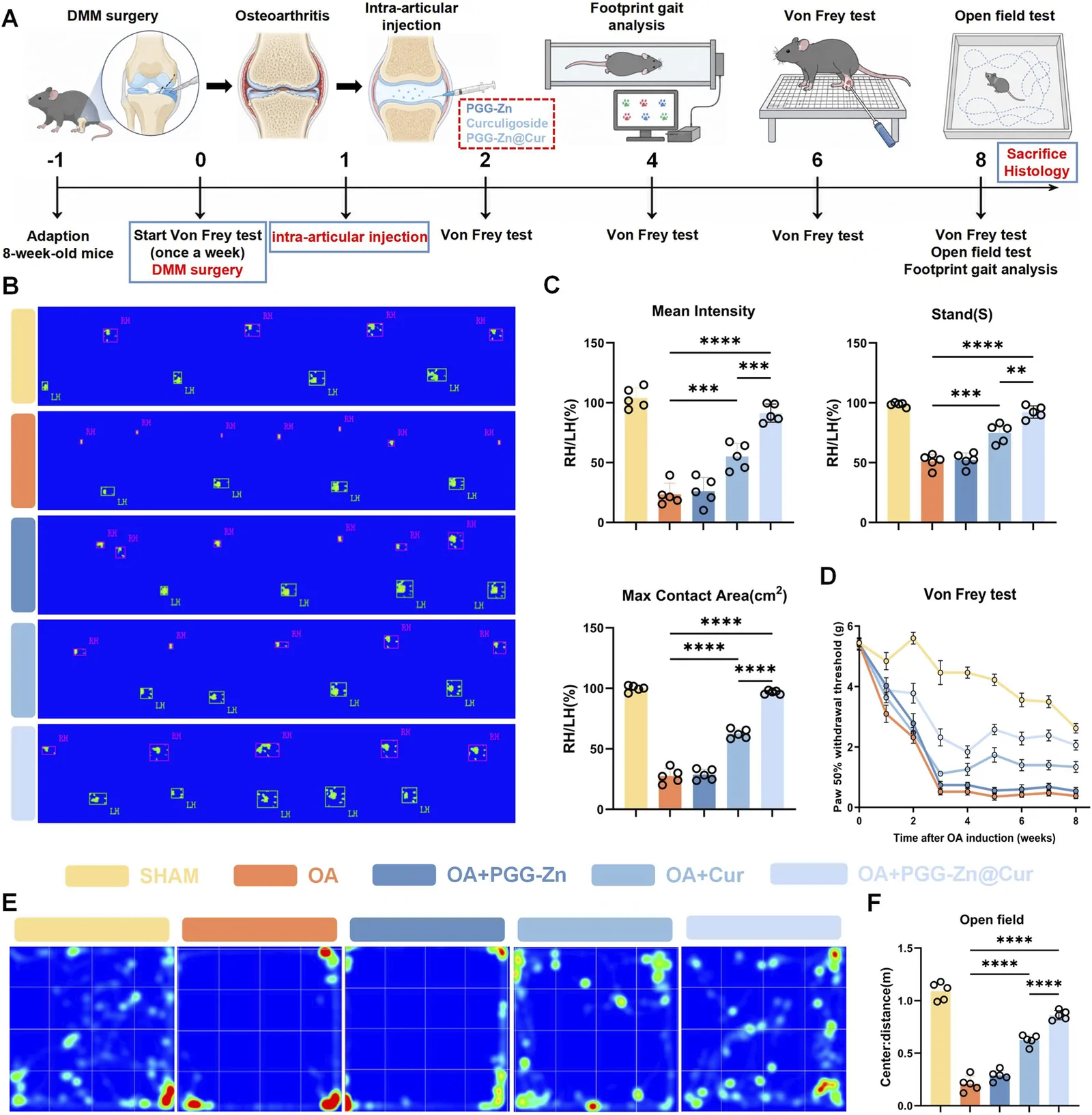
PGG-Zn@Cur improves gait symmetry and alleviates pain-related behavioral dysfunction in OA mice. **(A)** Schematic illustration of the *in vivo* experimental design. OA was induced by DMM surgery, followed by intra-articular administration of different formulations. Footprint gait analysis, Von Frey test, and open field test were performed before histological evaluation. **(B)** Representative footprint gait images from different treatment groups. RH and LH indicate right and left hindlimb footprints, respectively. **(C)** Quantitative analysis of gait-related parameters, including mean intensity, stand duration, and maximum contact area, expressed as RH/LH ratios. These parameters reflect hindlimb loading symmetry, stance stability, and paw-ground contact during locomotion. **(D)** Paw withdrawal threshold measured by the Von Frey test over time after OA induction. **(E)** Representative movement trajectory heatmaps from the open field test. **(F)** Quantitative analysis of center distance in the open field test, reflecting spontaneous exploratory activity. (*n* = 5 mice per group. *: *p* < 0.05; **: *p* < 0.01; ***: *p* < 0.001; ****: *p* < 0.0001; ns: not significant).

CatWalk XT gait analysis was first employed to assess OA-associated locomotor dysfunction. DMM-operated mice exhibited disrupted regularity in hindlimb gait patterns, whereas PGG-Zn@Cur treatment yielded more coordinated footprint distributions (Figure 5B). Quantitative analysis of the right hind/left hind (RH/LH) ratios for mean intensity, stance duration, and maximum contact area revealed significant deviations in the DMM group compared with the sham group, indicative of impaired hindlimb gait symmetry. Notably, while PGG-Zn alone failed to produce substantial improvements and free Cur conferred only partial functional benefits, PGG-Zn@Cur significantly restored hindlimb locomotor stability (Figure 5C). Mechanical allodynia was subsequently evaluated using the Von Frey up-down method. DMM-operated mice displayed a markedly reduced paw withdrawal threshold, reflecting mechanical hypersensitivity. PGG-Zn@Cur treatment significantly elevated the withdrawal threshold during the later observational period, whereas PGG-Zn alone exerted minimal modulatory effects and Cur monotherapy showed only partial amelioration (Figure 5D). Spontaneous exploratory activity was further assessed using the open field test. Representative locomotor trajectory heatmaps revealed decreased central zone exploration in DMM mice, which was notably increased following PGG-Zn@Cur administration (Figures 5E,F). Collectively, these findings demonstrate that PGG-Zn@Cur improves hindlimb gait symmetry, alleviates mechanical allodynia, and improved pain-related functional performance and exploratory behavior in DMM-operated mice, underscoring its potential as a disease-modifying therapeutic strategy for osteoarthritis.

### 3.6 PGG-Zn@Cur attenuated OA-associated cartilage degeneration and preserved matrix homeostasis

Following behavioral assessments, we further examined whether the functional improvement induced by PGG-Zn@Cur was accompanied by structural protection of articular cartilage. Safranin O/Fast Green staining was first performed to assess cartilage morphology and proteoglycan retention. The Sham group exhibited a smooth cartilage surface, preserved cartilage architecture, and intense Safranin O staining, indicating an intact proteoglycan-rich matrix. In contrast, the OA group showed typical degenerative features, including surface irregularity, matrix erosion, and reduced Safranin O staining. Cur partially preserved cartilage matrix staining. Notably, PGG-Zn@Cur more effectively maintained cartilage architecture and Safranin O-positive matrix, suggesting improved protection against OA-associated cartilage degeneration (Figure 6A).

**FIGURE 6:**
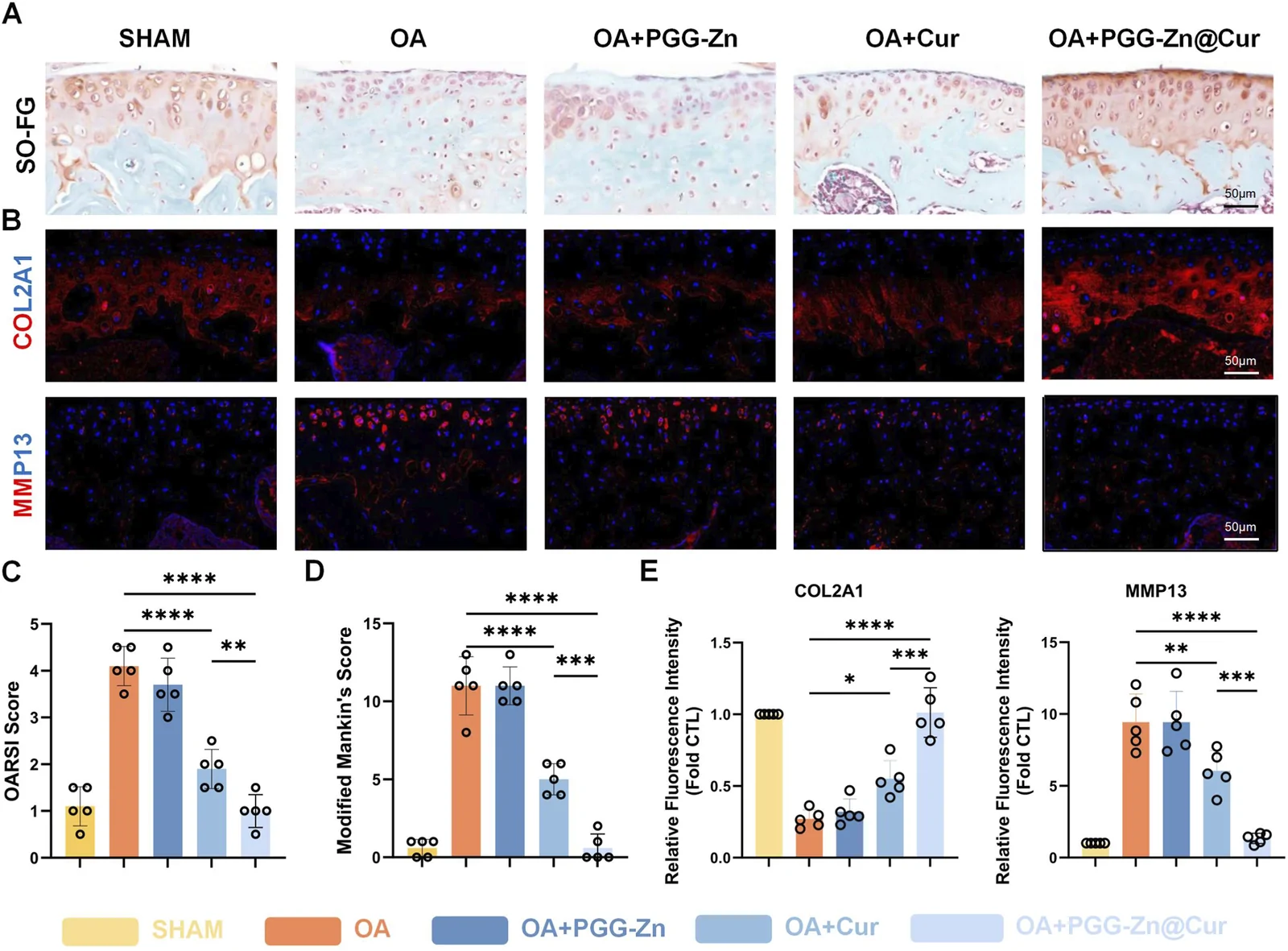
PGG-Zn@Cur attenuates OA-associated cartilage degeneration and preserves matrix homeostasis *in vivo*. **(A)** Representative Safranin O/Fast Green staining images of knee joint cartilage from the Sham, OA, OA+ PGG-Zn, OA+ Cur, and OA+ PGG-Zn@Cur groups. **(B)** Representative immunofluorescence images of COL2A1 and MMP13 in articular cartilage from different groups. Nuclei were counterstained with DAPI. **(C, D)** Quantitative analysis of cartilage degeneration using OARSI scores and modified Mankin’s scores. **(E)** Quantitative analysis of COL2A1 and MMP13 fluorescence intensity in articular cartilage. (*n* = 5 mice per group, Scale bar: 50 μm *: *p* < 0.05; **: *p* < 0.01; ***: *p* < 0.001; ****: *p* < 0.0001; ns: not significant).

OARSI and modified Mankin’s scores were then calculated to quantitatively evaluate cartilage degeneration. Consistent with the histological findings, PGG-Zn@Cur significantly decreased both OARSI and modified Mankin’s scores, indicating more pronounced attenuation of cartilage structural damage (Figures 6C,D).

Further IF staining of articular cartilage sections revealed markedly diminished COL2A1 fluorescence in the OA group, concomitant with enhanced MMP13-positive signals, indicative of augmented catabolic remodeling. Compared with either PGG-Zn or Cur monotherapy, PGG-Zn@Cur treatment more prominently restored COL2A1 expression and suppressed MMP13 accumulation (Figures 6B,E). These results demonstrate that PGG-Zn@Cur attenuates OA-associated cartilage degeneration, preserves proteoglycan-rich cartilage matrix, and maintains cartilage matrix metabolic homeostasis *in vivo*.

### 3.7 PGG-Zn@Cur attenuated PI3K-AKT pathway activation and apoptosis-associated cartilage damage

To further assess whether PI3K-AKT signaling was associated with the cartilage-preserving effect of PGG-Zn@Cur *in vivo*, the expression patterns of p-PI3K and p-AKT were evaluated in mouse articular cartilage. Compared with the sham group, DMM mice showed markedly stronger p-PI3K and p-AKT immunofluorescence signals, accompanied by a reduction in COL2A1 expression, suggesting enhanced PI3K-AKT pathway activity and cartilage matrix deterioration during OA development. Notably, PGG-Zn@Cur more effectively decreased p-PI3K and p-AKT fluorescence intensity while restoring COL2A1 expression in articular cartilage (Figures 7A,C).

**FIGURE 7:**
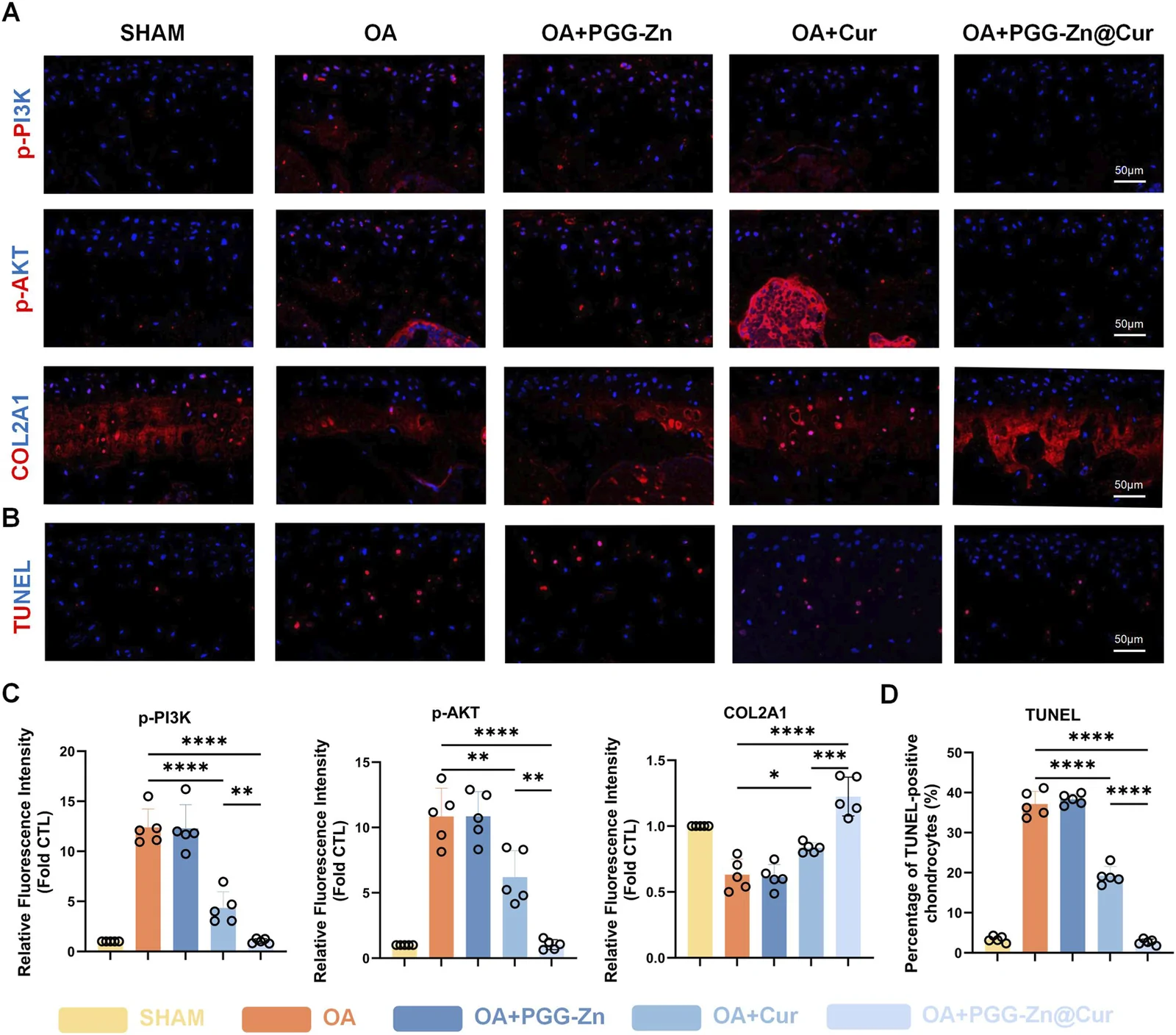
PGG-Zn@Cur attenuates PI3K-AKT pathway activation and apoptosis-associated cartilage damage *in vivo*. **(A)** Representative immunofluorescence images of p-PI3K, p-AKT, and COL2A1 in articular cartilage from the Sham, OA, OA+ PGG-Zn, OA+ Cur, and OA+ PGG-Zn@Cur groups. Nuclei were counterstained with DAPI. **(B)** Representative TUNEL staining images of articular cartilage from different groups. **(C)** Quantitative analysis of p-PI3K, p-AKT, and COL2A1 fluorescence intensity in articular cartilage. **(D)** Quantitative analysis of TUNEL-positive chondrocytes in articular cartilage. (*n* = 5 mice per group, Scale bar: 50 μm *: *p* < 0.05; **: *p* < 0.01; ***: *p* < 0.001; ****: *p* < 0.0001; ns: not significant).

TUNEL staining was further employed to evaluate chondrocyte apoptosis in articular cartilage sections. The DMM group exhibited a marked increase in TUNEL-positive cells, indicating enhanced apoptosis-associated cartilage damage during OA progression. Compared with the DMM group, PGG-Zn@Cur treatment significantly reduced the number of TUNEL-positive chondrocytes, whereas free Cur showed only a partial decreasing trend (Figures 7B,D). The *in vivo* results demonstrate that PGG-Zn@Cur attenuates OA-related PI3K-AKT phosphorylation signaling, preserves COL2A1 expression, and mitigates apoptosis-associated chondrocyte damage.

## 4 Discussion

Osteoarthritis (OA) is a degenerative joint disease characterized by progressive cartilage matrix degradation, pain-related dysfunction, and limited disease-modifying therapeutic options (Peng et al., 2025; Zhang Y. et al., 2026). Although intra-articular administration provides a direct route for local intervention, rapid drug clearance from the joint cavity and insufficient responsiveness to the pathological microenvironment remain major barriers to durable therapeutic efficacy (Bruno et al., 2022; Jiang and Zhang, 2023; Wu et al., 2025). In this study, we developed a Zn^2+^-coordinated PGG hydrogel microsphere system for Cur delivery. The resulting PGG-Zn@Cur microspheres displayed favorable microsphere morphology, efficient Cur loading, gradual degradation, and pH-responsive swelling and release behavior. Functionally, PGG-Zn@Cur showed good cytocompatibility, restored cartilage matrix metabolic homeostasis in IL-1β-stimulated chondrocytes, attenuated PI3K-AKT activation and apoptosis-associated responses *in vitro*, and further improved gait symmetry, mechanical hypersensitivity, cartilage degeneration, and apoptosis-related cartilage damage in OA mice. These findings suggest that PGG-Zn@Cur may serve as a local microenvironment-responsive delivery strategy for OA intervention.

Beyond serving as a passive drug reservoir, the PGG hydrogel possesses intrinsic functional attributes relevant to OA therapy. Gellan gum (GG), an anionic microbial polysaccharide, exhibits excellent biocompatibility and ion-responsive gelation, allowing the formation of hydrated networks that partially recapitulate the physicochemical features of cartilage extracellular matrix (ECM) (Alheib et al., 2022; Abreu et al., 2023). The incorporation of γ-polyglutamic acid (γ-PGA) further enriches the carboxylate density, generating a negatively charged, highly hydrated microenvironment that not only provides abundant coordination sites for metal ions but also mimics the anionic nature of aggrecan-rich cartilage matrix (Chiang et al., 2013). Consequently, the PGG composite may inherently support chondrocyte phenotype maintenance and ECM homeostasis through biomimetic hydration and charge distribution, independent of the loaded cargo.

Stimuli-responsive injectable hydrogels represent an important advancement by enabling spatiotemporally controlled drug release in response to pathological signals (Robby et al., 2025). The pH-responsive behavior of PGG-Zn@Cur provides an important material basis for adapting drug release to the OA-associated pathological microenvironment. During OA progression, inflammatory activation and metabolic remodeling can alter the joint microenvironment, including local acidification and accumulation of acidic metabolites (Fan X. et al., 2024; Dang et al., 2025). A delivery system that remains relatively stable under physiological conditions but undergoes network swelling in an acidic inflammatory microenvironment may help reduce premature drug leakage while promoting drug release at the diseased site (Zhang et al., 2022; Liao et al., 2024; Chen et al., 2025). In this study, PGG-Zn@Cur exhibited enhanced swelling and Cur release under acidic microenvironment-mimicking conditions, suggesting that the reversible Zn^2+^–carboxylate coordination network could respond to environmental changes. Such microenvironment-adaptive release may prolong intra-articular drug action, increase the effective exposure of chondrocytes to Cur, and thereby contribute to the suppression of inflammatory-catabolic responses, preservation of cartilage matrix homeostasis, and improvement of OA-associated functional impairment. Notably, beyond the acidic microenvironment, osteoarthritis drives robust accumulation of reactive oxygen species (ROS) and coenzyme A (CoA-SH), which markedly accelerates cartilage degeneration (Zahan et al., 2020). Numerous studies have focused on targeting these oxidative metabolites to achieve more site-specific delivery of biomaterials (Robby et al., 2024a). For instance, Robby et al. constructed a CoA-SH-responsive system by leveraging CoA-SH-triggered degradation of manganese dioxide (MnO_2_) (Robby et al., 2024b). In future work, we will design more precise delivery systems based on novel therapeutic targets to accomplish targeted OA treatment.

This sustained-release feature is particularly relevant for Cur delivery. Cur has been reported to possess anti-inflammatory, antioxidant, and tissue-protective activities, making it a potential therapeutic candidate for OA intervention (Wang et al., 2020; Du et al., 2025). However, the direct application of free Cur may be limited by unfavorable delivery-related properties, including limited solubility, rapid clearance, insufficient intra-articular retention, and uncontrolled drug exposure after injection. These limitations make it difficult to maintain an effective local drug concentration in articular cartilage for a prolonged period. Encapsulating Cur into PGG-Zn microspheres provides a strategy to improve its local availability and achieve sustained release. In this sense, PGG-Zn@Cur primarily functions as a sustained-release intra-articular delivery system, allowing Cur to exert prolonged biological effects on chondrocytes and cartilage tissue.

Beyond drug encapsulation, PGG-Zn hydrogel microspheres are also applicable for localized gene-modified therapy. Lentiviruses and adeno-associated viruses, despite their widespread use in gene therapy research, suffer from high immunogenicity, rapid *in vivo* clearance, and lack of stimulus-responsive release, all of which contribute to low transfection efficiency and hinder clinical translation. The combination of biomaterials with genetic modification has emerged as a compelling alternative. For instance, Song et al. demonstrated that heparin-modified ACBP nanoparticles delivering siFoxM1 effectively ameliorated cartilage degeneration in DMM mice by targeting the acetyl-CoA–FOXM1 senescence axis (Song et al., 2023). Our PGG-Zn microspheres present distinct advantages for gene delivery: the anionic microbial polysaccharide PGG ensures favorable biocompatibility and low immunogenicity, while pH-responsive degradation enables targeted cargo release within the inflamed OA joint microenvironment, which is anticipated to substantially improve plasmid transfection efficiency. Future work will focus on experimentally validating the gene delivery capacity of PGG-Zn microspheres and exploring combined drug-gene therapeutic strategies for enhanced OA treatment.

Chondrocyte survival and matrix homeostasis are central to the maintenance of articular cartilage integrity (Fan Y. et al., 2024). During OA progression, persistent inflammatory stimulation and mechanical stress disturb the balance between cell survival and matrix turnover, leading to progressive cartilage degeneration (De Roover et al., 2023; Gan et al., 2024). Among the intracellular signaling networks involved in this process, PI3K-AKT signaling represents an important regulatory axis linking inflammatory stress, apoptosis-related injury, and cartilage matrix remodeling (Sun et al., 2020; Xu et al., 2021). Beyond cell survival, AKT can phosphorylate the IKK complex to activate NF-κB and promote p65 nuclear translocation, amplifying inflammatory-catabolic responses in chronic inflammation (Guo et al., 2024). Lu et al. showed that PI3K/AKT/NF-κB and Nrf2/ROS/Bax signaling participate in IL-1β-induced inflammation, oxidative stress, and chondrocyte apoptosis (Lu et al., 2021). While Wang et al. linked the TLR4-RAC1-PIK3CA-AKT-NF-κB axis to cartilage degeneration and subchondral bone changes (Wang L. et al., 2022). Thus, aberrant PI3K-AKT activation and its coupling with IKK/NF-κB in the OA microenvironment may drive iNOS, MMP13, and ADAMTS5 expression, exacerbating matrix destruction. Here, PGG-Zn@Cur attenuated IL-1β-induced PI3K-AKT activation and apoptosis-related injury, suggesting it may disrupt the AKT-centered inflammatory stress network to dampen downstream catabolic and apoptotic responses. However, the precise interplay between PI3K-AKT and IKK/NF-κB modules requires further investigation through IKK phosphorylation, IκBα stability, p65 translocation, and pathway-specific inhibition studies.

Several limitations should be acknowledged. First, although PGG-Zn@Cur showed pH-responsive swelling and release behavior *in vitro*, the dynamic changes in intra-articular pH and lactate levels during OA progression were not directly measured in this study. Future studies should characterize the local metabolic microenvironment of OA joints to better define the relationship between pathological biochemical cues and microsphere release behavior. Secondly, chondroitin sulfate is endogenously present in cartilage tissue. Its sulfate and carboxyl groups may interact with zinc ions, thereby interfering with Cur release. However, chondroitin sulfate possesses a larger molecular volume compared with lactic acid molecules, which hinders its infiltration and binding to PGG-Zn hydrogel microspheres. Further experimental investigations are required in future studies to rule out such interfering effects. Thirdly the present study mainly focused on chondrocytes and articular cartilage, whereas OA is a whole-joint disease involving cartilage, synovium, subchondral bone, meniscus, immune cells, and sensory innervation. Future investigations should further assess the influence of PGG-Zn@Cur on the broader joint microenvironment and disease progression.

In summary, we engineered Cur-loaded PGG-Zn hydrogel microspheres (PGG-Zn@Cur) via Zn^2+^-mediated dynamic coordination crosslinking for localized OA intervention. Our findings demonstrate that PGG-Zn@Cur exhibits excellent cytocompatibility and restores cartilage matrix metabolic homeostasis in IL-1β-stimulated chondrocytes. *In vivo*, repeated intra-articular administration of PGG-Zn@Cur improved pain-related functional outcomes and attenuated DMM-induced cartilage degeneration. Mechanistically, PGG-Zn@Cur preserves cartilage matrix integrity associated with PI3K-AKT signaling and downstream apoptotic cascades, suggesting that this microenvironment-responsive delivery system holds considerable potential as a localized therapeutic strategy for osteoarthritis.

## Data Availability

The transcriptomic sequencing data presented in this study have been deposited in the NCBI Gene Expression Omnibus (GEO) repository under accession number GSE345836.

## References

[B1] Abreu C. M. Lago M. E. L. Pires J. Reis R. L. da Silva L. P. Marques A. P. (2023). Gellan gum-based hydrogels support the recreation of the dermal papilla microenvironment. Biomater. Adv. 150, 213437. 10.1016/j.bioadv.2023.213437 37116455

[B2] Alheib O. da Silva L. P. da Silva Morais A. Mesquita K. A. Pirraco R. P. Reis R. L. (2022). Injectable laminin-biofunctionalized gellan gum hydrogels loaded with myoblasts for skeletal muscle regeneration. Acta Biomater. 143, 282–294. 10.1016/j.actbio.2022.03.008 35278687

[B3] Bharadwaj U. U. Lynch J. A. Joseph G. B. Akkaya Z. Nevitt M. C. Lane N. E. (2025). Intra-articular knee injections and progression of knee osteoarthritis: data from the osteoarthritis initiative. Radiology 315, e233081. 10.1148/radiol.233081 40423542 PMC12127947

[B4] Biswal S. Parmanik A. Das D. Sahoo R. N. Nayak A. K. (2025). Gellan gum-based *in-situ* gel formulations for ocular drug delivery: a practical approach. Int. J. Biol. Macromol. 290, 138979. 10.1016/j.ijbiomac.2024.138979 39708866

[B5] Bruno M. C. Cristiano M. C. Celia C. d’Avanzo N. Mancuso A. Paolino D. (2022). Injectable drug delivery systems for osteoarthritis and rheumatoid arthritis. ACS Nano 16, 19665–19690. 10.1021/acsnano.2c06393 36512378

[B6] Chen J. Deng M. Wang J. Liu Y. Hu Z. Luan F. (2025). Recent advances in injectable hydrogels for osteoarthritis treatments. Front. Bioeng. Biotechnol. 13, 1644222. 10.3389/fbioe.2025.1644222 40843442 PMC12364895

[B7] Chiang W.-H. Huang W.-C. Chang C.-W. Shen M.-Y. Shih Z.-F. Huang Y.-F. (2013). Functionalized polymersomes with outlayered polyelectrolyte gels for potential tumor-targeted delivery of multimodal therapies and MR imaging. J. Control Release 168, 280–288. 10.1016/j.jconrel.2013.03.029 23562635

[B8] Dang Y. Liu Y. Zhang B. Zhang X. (2025). Aging microenvironment in osteoarthritis focusing on early-stage alterations and targeted therapies. Bone Res. 13, 84. 10.1038/s41413-025-00465-6 41073390 PMC12514018

[B9] De Roover A. Escribano-Núñez A. Monteagudo S. Lories R. (2023). Fundamentals of osteoarthritis: inflammatory mediators in osteoarthritis. Osteoarthr. Cartil. 31, 1303–1311. 10.1016/j.joca.2023.06.005 37353140

[B10] Du J. Qin W. Wen F. Liu Y. Zhang Q. Liu W. (2025). Curculigoside is a promising osteoprotective agent for osteoporosis: review. Drug Des. Devel Ther. 19, 3323–3336. 10.2147/DDDT.S519174 40322028 PMC12048297

[B11] Fan X. Sun A. R. Young R. S. E. Afara I. O. Hamilton B. R. Ong L. J. Y. (2024a). Spatial analysis of the osteoarthritis microenvironment: techniques, insights, and applications. Bone Res. 12, 7. 10.1038/s41413-023-00304-6 PMC10838951 38311627

[B12] Fan Y. Bian X. Meng X. Li L. Fu L. Zhang Y. (2024b). Unveiling inflammatory and prehypertrophic cell populations as key contributors to knee cartilage degeneration in osteoarthritis using multi-omics data integration. Ann. Rheum. Dis. 83, 926–944. 10.1136/ard-2023-224420 38325908 PMC11187367

[B13] Feng Q. Xu R. Niu P. Tian X. Chang M. (2026). Temporal trends and sociodemographic disparities in osteoarthritis prevalence among U.S. adults. TransMed 1, 100017. 10.1016/j.tmed.2026.100017

[B14] Gambaro F. M. Ummarino A. Torres Andón F. Ronzoni F. Di Matteo B. Kon E. (2021). Drug delivery systems for the treatment of knee osteoarthritis: a systematic review of *in vivo* studies. Int. J. Mol. Sci. 22, 9137. 10.3390/ijms22179137 PMC8431358 34502046

[B15] Gan D. Tao C. Jin X. Wu X. Yan Q. Zhong Y. (2024). Piezo1 activation accelerates osteoarthritis progression and the targeted therapy effect of artemisinin. J. Adv. Res. 62, 105–117. 10.1016/j.jare.2023.09.040 37758057 PMC11331168

[B16] Glasson S. S. Chambers M. G. Van Den Berg W. B. Little C. B. (2010). The OARSI histopathology initiative - recommendations for histological assessments of osteoarthritis in the mouse. Osteoarthr. Cartil. 18 (Suppl. 3), S17–S23. 10.1016/j.joca.2010.05.025 20864019

[B17] Gong Y. Wang P. Cao R. Wu J. Ji H. Wang M. (2023). Exudate absorbing and antimicrobial hydrogel integrated with multifunctional curcumin-loaded magnesium polyphenol network for facilitating burn wound healing. ACS Nano 17, 22355–22370. 10.1021/acsnano.3c04556 37930078

[B18] Guo Q. Jin Y. Chen X. Ye X. Shen X. Lin M. (2024). NF-κB in biology and targeted therapy: new insights and translational implications. Signal Transduct. Target Ther. 9, 53. 10.1038/s41392-024-01757-9 PMC10910037 38433280

[B19] Jiang Q. Zhang S. (2023). Stimulus-responsive drug delivery nanoplatforms for osteoarthritis therapy. Small 19, e2206929. 10.1002/smll.202206929 36905239

[B20] Jin R. Li J. Huang B. Lu J. Huang J. Wu L. (2024). Amelioration of osteoarthritis through salicylic acid nano-formulated self-therapeutic prodrug for the prolonged launch of salicylic acid to damaged cartilage. Appl. Mater. Today 37, 102102. 10.1016/j.apmt.2024.102102

[B21] Kumavat R. Kumar V. Malhotra R. Pandit H. Jones E. Ponchel F. (2021). Biomarkers of joint damage in osteoarthritis: current status and future directions. Mediat. Inflamm. 2021, 5574582. 10.1155/2021/5574582 33776572 PMC7969115

[B22] Lei L. Wang H. Zhao Z. Huang Y. Huang X. Guo X. (2025). Curculigoside upregulates BMAL1 to decrease nucleus pulposus cell apoptosis by inhibiting the JAK/STAT3 pathway. Osteoarthr. Cartil. 33, 412–425. 10.1016/j.joca.2024.11.009 39622432

[B23] Li G. Liu S. Chen Y. Zhao J. Xu H. Weng J. (2023). An injectable liposome-anchored teriparatide incorporated gallic acid-grafted gelatin hydrogel for osteoarthritis treatment. Nat. Commun. 14, 3159. 10.1038/s41467-023-38597-0 37258510 PMC10232438

[B24] Liao S. Jia S. Yue Y. Zeng H. Lin J. Liu P. (2024). Advancements in pH-Responsive nanoparticles for osteoarthritis treatment: opportunities and challenges. Front. Bioeng. Biotechnol. 12, 1426794. 10.3389/fbioe.2024.1426794 39036562 PMC11260422

[B25] Liu Y. Hou M. Pan Z. Tian X. Zhao Z. Liu T. (2022). Arctiin-reinforced antioxidant microcarrier antagonizes osteoarthritis progression. J. Nanobiotechnology 20, 303. 10.1186/s12951-022-01505-7 PMC9235181 35761235

[B26] Liu X. Ma B. Hu S. Li D. Pan C. Xu Z. (2025). Phase-adapted metal ion supply for spinal cord repair with a Mg-Zn incorporated chimeric microsphere. Biomaterials 320, 123253. 10.1016/j.biomaterials.2025.123253 40107180

[B27] Lu H. Fu C. Kong S. Wang X. Sun L. Lin Z. (2021). Maltol prevents the progression of osteoarthritis by targeting PI3K/Akt/NF-κB pathway: *in vitro* and *in vivo* studies. J. Cell Mol. Med. 25, 499–509. 10.1111/jcmm.16104 33211383 PMC7810946

[B28] Öztürk K. Kaplan M. Çalış S. (2024). Effects of nanoparticle size, shape, and zeta potential on drug delivery. Int. J. Pharm. 666, 124799. 10.1016/j.ijpharm.2024.124799 39369767

[B29] Park J. Ghanim R. Rahematpura A. Gerage C. Abramson A. (2024). Electromechanical convective drug delivery devices for overcoming diffusion barriers. J. Control Release 366, 650–667. 10.1016/j.jconrel.2024.01.008 38190971

[B30] Peng X. Chen X. Zhang Y. Tian Z. Wang M. Chen Z. (2025). Advances in the pathology and treatment of osteoarthritis. J. Adv. Res. 78, 257–283. 10.1016/j.jare.2025.01.053 39889821 PMC12684957

[B31] Pritzker K. P. H. Gay S. Jimenez S. A. Ostergaard K. Pelletier J.-P. Revell P. A. (2006). Osteoarthritis cartilage histopathology: grading and staging. Osteoarthr. Cartil. 14, 13–29. 10.1016/j.joca.2005.07.014 16242352

[B32] Qian Y. Wang S. Sha Y. Wu J. Chi Q. Huang L. (2026). Hydrogel-guided p-LOX nanodrug delivery modulates metabolic microenvironment for cartilage repair in osteoarthritis. Biomaterials 328, 123806. 10.1016/j.biomaterials.2025.123806 41177139

[B33] Robby A. I. Jiang S. Jin E.-J. Park S. Y. (2024a). Coenzyme-A-Responsive nanogel-coated electrochemical sensor for osteoarthritis-detection-based genetic models. Gels 10, 451. 10.3390/gels10070451 39057474 PMC11276253

[B34] Robby A. I. Jiang S. Jin E.-J. Park S. Y. (2024b). Electrochemical and fluorescence MnO2-Polymer dot electrode sensor for osteoarthritis-based peroxisomal β-Oxidation knockout model. Biosens. (Basel) 14, 357. 10.3390/bios14070357 PMC11275033 39056633

[B35] Robby A. I. Kim E. H. Huh K. M. Jin E.-J. Park K. D. Park S. Y. (2025). Mevalonate pathway-triggered phase transition of injectable hydrogel for cholesterol-downregulated therapy of osteoarthritis. Bioact. Mater 51, 876–888. 10.1016/j.bioactmat.2025.06.047 40678261 PMC12269283

[B36] Song J. Kim E. H. Yang J.-H. Kim D. Robby A. I. Kim S. (2023). Upregulated FOXM1 stimulates chondrocyte senescence in Acot12^-/-^ Nudt7^-/-^ double knockout mice. Theranostics 13, 5207–5222. 10.7150/thno.89033 37908734 PMC10614692

[B37] Sun K. Luo J. Guo J. Yao X. Jing X. Guo F. (2020). The PI3K/AKT/mTOR signaling pathway in osteoarthritis: a narrative review. Osteoarthr. Cartil. 28, 400–409. 10.1016/j.joca.2020.02.027 32081707

[B38] Tang C. Wang H. Guo L. Cui Y. Zou C. Hu J. (2025). Multifunctional nanomedicine for targeted atherosclerosis therapy: activating plaque clearance Cascade and suppressing inflammation. ACS Nano 19, 3339–3361. 10.1021/acsnano.4c12131 39812806

[B39] Wang S. Liu W. Wang J. Bai X. (2020). Curculigoside inhibits ferroptosis in ulcerative colitis through the induction of GPX4. Life Sci. 259, 118356. 10.1016/j.lfs.2020.118356 32861798

[B40] Wang L. Xu P. Xu Y. Cui R. Yang Y. Zou Z. (2022). A discovery of clinically approved panlongqi tablet for repositioning to treat osteoarthritis by inhibiting PI3K/AKT activation. Phytomedicine 105, 154360. 10.1016/j.phymed.2022.154360 35964457

[B41] Wang M. M. Tan G. Jiang H. Liu A. Wu R. Li J. (2022). Molecular crosstalk between articular cartilage, meniscus, synovium, and subchondral bone in osteoarthritis. Bone Jt. Res. 11, 862–872. 10.1302/2046-3758.1112.BJR-2022-0215.R1 36464496 PMC9792876

[B42] Wu M. Chen Z. Song B. Wang X. Liang W. (2025). Current status and future perspectives of research on intra-articular drug delivery systems for osteoarthritis therapy. Acta Biomater. 203, 59–77. 10.1016/j.actbio.2025.07.057 40721187

[B43] Wu J. Liu Y. Shi K. Huang H. Qian Z. (2026). Harnessing biophysical stimuli to regulate bone remodeling: emerging therapeutic strategies and advances in regenerative engineering. EngMedicine 3, 100138. 10.1016/j.engmed.2026.100138

[B44] Xu K. He Y. Moqbel S. A. A. Zhou X. Wu L. Bao J. (2021). SIRT3 ameliorates osteoarthritis *via* regulating chondrocyte autophagy and apoptosis through the PI3K/Akt/mTOR pathway. Int. J. Biol. Macromol. 175, 351–360. 10.1016/j.ijbiomac.2021.02.029 33556400

[B45] Xu Y. Wen K. Liu A. Wang X. Xu H. Wen H. (2023). Efficacy of curculigoside in protecting against ischemic brain injury through regulation of oxidative stress and NF-κB and PI3K/Akt expression. J. Ethnopharmacol. 301, 115804. 10.1016/j.jep.2022.115804 36228892

[B46] Yang Y. Wang P. Gong Y. Yu Z. Gan Y. Li P. (2023). Curcumin-zinc framework encapsulated microneedle patch for promoting hair growth. Theranostics 13, 3675–3688. 10.7150/thno.84118 37441591 PMC10334826

[B47] Yang B. Li Z. Yang Z. Zhao P. Lin S. Wu J. (2025). Recapitulating hypoxic metabolism in cartilaginous organoids via adaptive cell-matrix interactions enhances histone lactylation and cartilage regeneration. Nat. Commun. 16, 2711. 10.1038/s41467-025-57779-6 40108220 PMC11923171

[B48] Yao Q. Wu X. Tao C. Gong W. Chen M. Qu M. (2023). Osteoarthritis: pathogenic signaling pathways and therapeutic targets. Signal Transduct. Target Ther. 8, 56. 10.1038/s41392-023-01330-w 36737426 PMC9898571

[B49] Zahan O.-M. Serban O. Gherman C. Fodor D. (2020). The evaluation of oxidative stress in osteoarthritis. Med. Pharm. Rep. 93, 12–22. 10.15386/mpr-1422 32133442 PMC7051818

[B50] Zhang M. Hu W. Cai C. Wu Y. Li J. Dong S. (2022). Advanced application of stimuli-responsive drug delivery system for inflammatory arthritis treatment. Mater Today Bio 14, 100223. 10.1016/j.mtbio.2022.100223 35243298 PMC8881671

[B51] Zhang X. Zhou H.-F. Chen J.-Y. Deng J.-H. Zhou Z.-M. Tang H.-X. (2026a). Resveratrol in degenerative musculoskeletal diseases: a homology of medicine and food perspective. Food & Med. Homol. 3, 9420093. 10.26599/FMH.2026.9420093

[B52] Zhang Y. Han Y. Sun Y. Hao L. Gao Y. Ye J. (2026b). Osteoarthritis: molecular pathogenesis and potential therapeutic options. Signal Transduct. Target Ther. 11, 81. 10.1038/s41392-025-02556-6 PMC12960730 41781374

[B53] Zhou J. Liang J. Yang K. Zhang G. (2026). Injectable hydrogels loaded with EGCG/KGN for the treatment of osteoarthritis. J. Biomater. Appl. 40, 819–829. 10.1177/08853282251379151 40939028

[B54] Zou F. Zhang K. Sun X. Cheng X. Wang T. Kuang B. (2026). Inhibiting Arachidonate-5-lipoxygenase expression ameliorates osteoarthritis progression by suppressing ferroptosis via the JAK2/STAT3 signaling pathway. Free Radic. Biol. Med. 246, 334–349. 10.1016/j.freeradbiomed.2026.01.035 41581577

