# Supplementary material for: pH-responsive Zn2+-coordinated gellan gum/γ-polyglutamic acid hydrogel microspheres as functional biomaterials for intra-articular delivery: physicochemical design, smart release, and chondroprotective performance in osteoarthritis
Source: Front Bioeng Biotechnol. 2026 Sep 29;14:1924086. doi: 10.3389/fbioe.2026.1924086 (PMC13658529; doi:10.3389/fbioe.2026.1924086)
Supplement: Supplementary file 1 [file Supplementaryfile1.docx]

**Supplementary Figure 1**

**Supplementary Figure 1. pH-dependent zeta potential of PGG-Zn@Cur microspheres.** The zeta potential of PGG-Zn@Cur microspheres was measured at pH 5.5, 7.4, and 9.5. The microspheres exhibited negative zeta potentials under all tested conditions, with increasingly negative values at higher pH. Data are presented as the mean ± SD (n = 3 independent samples).
